# Hepatocellular carcinoma after a sustained virological response by direct‐acting antivirals harbors *TP53* inactivation

**DOI:** 10.1002/cam4.4571

**Published:** 2022-02-17

**Authors:** Taisuke Imamura, Yukiyasu Okamura, Keiichi Ohshima, Katsuhiko Uesaka, Teiichi Sugiura, Takaaki Ito, Yusuke Yamamoto, Ryo Ashida, Katsuhisa Ohgi, Shimpei Otsuka, Sumiko Ohnami, Takeshi Nagashima, Keiichi Hatakeyama, Yuko Kakuda, Takashi Sugino, Kenichi Urakami, Yasuto Akiyama, Ken Yamaguchi

**Affiliations:** ^1^ Division of Hepato‐Biliary‐Pancreatic Surgery Shizuoka Cancer Center Shizuoka Japan; ^2^ Department of Digestive Surgery Nihon University School of Medicine Tokyo Japan; ^3^ Medical Genetics Division Shizuoka Cancer Center Research Institute Shizuoka Japan; ^4^ Cancer Diagnostics Research Division Shizuoka Cancer Center Research Institute Shizuoka Japan; ^5^ SRL, Inc. Tokyo Japan; ^6^ Division of Pathology Shizuoka Cancer Center Shizuoka Japan; ^7^ Immunotherapy Division Shizuoka Cancer Center Research Institute Shizuoka Japan; ^8^ Shizuoka Cancer Center Hospital and Research Institute Shizuoka Japan

**Keywords:** direct‐acting antiviral agent, hepatitis C virus, hepatocellular carcinoma, interferon, sustained virological response

## Abstract

**Introduction:**

The genomic characteristics of hepatocellular carcinoma (HCC) after a sustained virological response (SVR) and its differences according to whether an SVR was achieved by treatment with direct‐acting antivirals (DAA) or interferon (IFN) are still not fully understood.

**Methods:**

Sixty‐nine surgically resected HCCs from patients with hepatitis C virus infection were analyzed by gene expression profiling and whole‐exome sequencing.

**Results:**

Among the 69 HCC patients, 34 HCCs in which an SVR was not achieved at the time of surgery were classified as HCV‐positive, and 35 HCCs in which an SVR was achieved at the time of surgery were classified as HCV‐SVR. According to the HCV treatment, 35 HCV‐SVR HCCs were classified into two groups: eight tumors with DAA (HCV‐SVR‐DAA) and 24 tumors with interferon (HCV‐SVR‐IFN). The frequency of samples with *ARID2* mutations was significantly lower in HCV‐SVR than in HCV‐positive tumors (*p* = 0.048). In contrast, the frequency of samples with *PREX2* mutations was significantly higher in HCV‐SVR samples than in HCV‐positive samples (*p* = 0.048). Among the patients with HCV‐SVR, the frequency of samples with *TP53* mutations was significantly higher in HCV‐SVR‐DAA tumors than in HCV‐SVR‐IFN tumors (*p* = 0.030). *TP53* inactivation scores in HCV‐SVR‐DAA tumors were found to be significantly enhanced in comparison to HCV‐SVR‐IFN tumors (*p* = 0.022). In addition, chromosomal instability and PI3K/AKT/mTOR pathway signatures were enhanced in HCV‐SVR‐DAA tumors. HCV‐SVR‐DAA was significantly associated with portal vein invasion (*p* = 0.003) in comparison to HCV‐SVR‐IFN.

**Conclusion:**

Our dataset potentially serves as a fundamental resource for the genomic characteristics of HCV‐SVR‐DAA tumors. Our comprehensive genetic profiling by WES revealed significant differences in the mutation rate of several driver genes between HCV‐positive tumors and HCV‐SVR tumors. Furthermore, it was revealed that the frequency of samples with mutations in *TP53* was significantly higher in HCV‐SVR‐DAA tumors than in HCV‐SVR‐IFN tumors.

## Graphical abstract

Our dataset potentially serves as a fundamental resource for the optimal management of HCC using the genomic information of HCV‐SVR‐DAA patients. The frequency of samples with TP53 mutations was significantly higher in HCV‐SVR‐DAA tumors than in HCV‐SVR‐IFN tumors.

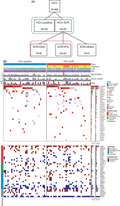

## 1 INTRODUCTION

Worldwide, hepatocellular carcinoma (HCC) is the fifth‐most common type of cancer and is the second leading cause of cancer death, and its incidence has been increasing.^1^ At the time of writing this report, surgical resection or liver transplantation are the only effective treatment options. However, no clinical molecular markers are available for the early diagnosis of HCC and there are few approved targeted molecular therapies.^2^ Accordingly, patient outcomes remain unsatisfactory.

HCC is frequently caused by hepatitis C virus (HCV) infection. The advent of direct‐acting antiviral (DAA) therapy represented a major advance in the treatment of HCV. Treatment with new interferon (IFN)‐free DAA therapies has achieved sustained virological response (SVR) rates of >90%.^3^ Although an SVR can reduce the overall risk of HCC in HCV patients by >70%,^4^ an SVR is insufficient to eliminate the risk of HCC occurring on the background of HCV infection, especially in cases with severe liver fibrosis.^5^, ^6^

The pathogenesis of HCC caused by HCV infection and the risk of HCC after curative treatment with DAAs have not been completely elucidated.^7^ As an RNA virus that has little potential to integrate its genetic material into the host genome, the contribution of HCV to hepatocarcinogenesis may be due to chronic infection‐driven inflammation of the liver in addition to the progression of liver fibrosis with the formation of a carcinogenic microenvironment.^7^, ^8^ Recently, the number of cases in which surgical resection was performed for HCC, even following the achievement of an SVR with DAA therapy, as well as the opportunities to obtain HCC specimens after the achievement of an SVR by DAA therapy has been increasing. Indeed, different clinical characteristics of HCC—even following an SVR—from those of HCC without an SVR have been reported,^9^, ^10^, ^11^, ^12^, ^13^ indicating that there might be alterations in the genomic features and tumor microenvironment after treatment. We assessed the variation in the genomic spectra between the HCV status and treatment for HCV using whole‐exome sequencing (WES) and gene expression profiling (GEP) and identified molecular alterations that may represent potential therapeutic targets or novel biomarkers.

## 2 METHODS

### 2.1 Ethics statement

The High‐tech Omics‐based Patient Evaluation (HOPE) project was initiated by Shizuoka Cancer Center in order to investigate the biological characteristics of cancer and predisposing factors of cancer patients.^14^ With the aim of advancing precision medicine, various types of cancer were subjected to multiomics‐based analyses. The HOPE project was conducted in accordance with the Ethical Guidelines for Human Genome and Genetic Analysis Research, revised in 2013.^14^ All patients gave their written informed consent for participation in the project. Our study used data obtained by the HOPE project and received approval from the institutional review board of Shizuoka Cancer Center (approval no. 25–33, and 202–22‐2020‐1‐2). The study was performed in conformance with the Declaration of Helsinki.

### 2.2 Patient selection and study design

Patients who had undergone surgical resection for the treatment of cancer at Shizuoka Cancer Center Hospital, and for whom an adequate amount of fresh cancer tissue was available were considered as candidates for the HOPE project. A total of 69 HCCs from patients with HCV infection, who were managed from January 2014 to March 2019, were included in the analysis of the HOPE project. SVR is defined as the absence of viremia at 24 weeks after the cessation of all antiviral medications. HCCs in which an SVR had been achieved at the time of surgery were classified as HCV‐SVR, and HCCs in which an SVR was not achieved at the time of surgery—despite the presence or absence of treatment for HCV—were classified as HCV‐positive. Patients in whom an SVR was achieved with DAA therapy were classified into the HCV‐SVR‐DAA group and those in whom an SVR was achieved with IFN were classified into the HCV‐SVR‐IFN group. The DAAs administered to patients in the DAA group included combination therapy of sofosbuvir and ledipasvir (*n* = 3), combination therapy with asunaprevir and daclatasvir (*n* = 3), and monotherapy of sofosbuvir (*n* = 2). All tumor specimens received a pathological diagnosis of HCC. A retrospective analysis was performed to investigate the clinicopathological and genomic factors of the tumors. To validate the prognostic impact of the gene expression and mutations in the TCGA cohort, mutation data and clinical data were extracted from previous articles^15^ and an online portal (https://www.cbioportal.org). Expression profiles were extracted from a public database (https://www.proteinatlas.org). The sample list used in this study is provided in Table S1. All patients enrolled in the present study were Asian, while in the TCGA, 36.3% of patients were Asian and the largest population was White (41.5%). The clinical stage in the TCGA cohort was more advanced (Stage I/II/III/IV, 49%/25%/24%/2%) in comparison to the present study cohort (Stage I/II/III/IV, 77%/16%/7%/0%).

### 2.3 Clinical samples

We prepared tumor tissue samples from fresh surgical specimens. Approximately ≥0.1 g of cancer tissue was needed for the examination. We also obtained the surrounding normal tissue. In addition, we collected peripheral blood as a control for whole‐exome sequencing (WES). For the DNA analysis, dissected tissue and blood samples were immediately snap‐frozen in liquid nitrogen prior to extraction of DNA. The extraction of DNA from tissue samples was performed using a QIAmp DNA Blood Mini Kit (Qiagen, Venlo, Netherlands). Quantification of DNA was performed using a NanoDrop and Qubit 2.0 Fluorometer (Thermo Fisher Scientific, Waltham, MA, USA). For the analysis of RNA, tissue samples were immersed in RNAlater solution (Thermo Fisher Scientific), then minced, and stored overnight at 4°C prior to the extraction of RNA.

### 2.4 Detection of somatic mutations

The kit used in WES supplies 292903 amplicons to amplify the exons of 18835 genes. These amplicons account for 57.7 Mb of the human genome, and 34.8 Mb of the exons of RefSeq genes. In total, 1.7 Mb of the human genome is encompassed by these amplicons of 1.3 Mb overlapping exons of RefSeq genes. Binary raw data derived from the semiconductor DNA sequencer were converted into sequence reads using the Torrent Suite software program (ver. 4.4, Thermo Fisher Scientific) that were mapped to the reference human genome (UCSC hg19). At this step, sequence data derived from tumor and blood samples were individually analyzed, and mapping results were saved as BAM files. Two BAM files were uploaded to the Ion Reporter system (ver. 4.4, Thermo Fisher Scientific) and analyzed concurrently with the AmpliSeq exome tumor‐normal pair workflow. In this study, the WES analysis focused on single nucleotide variants (SNVs) and short insertions and deletions (indels) in an exon and splice site for somatic mutations.

### 2.5 Filtering of detected mutations

The list of mutations obtained through the aforementioned procedure was filtered to discard false‐positive findings. Mutations fulfilling at least one of following criteria were eliminated:

1. Quality score <50
2. Depth of coverage <20 (for SNVs), <50 (for indels)
3. Variant allele frequency in matched controls >2.5% (for SNVs), >0% (for indels)
4. Variant reads observed in either forward or reverse strands but not both
5. Clipped sequence length <90 (avg_clipped_length <90)
6. Variant is located at the sequence end (avg_pos_as_fraction <0.05)
7. MAPQ1/MAPQ0 read ratio <0.8
8. Difference between reference and variant bases of read length >80
9. Variants enriched in MAPQ0 reads (*p*‐value <0.1 by Fisher's exact test)
10. Mutation matches with the in‐house false‐positive list

MAPQ1 and MAPQ0 reads represent the number of reads with a mapping quality of ≥1 and 0, respectively. Parameters specified in criteria^5^, ^6^, ^7^, ^8^, ^9^ were determined via bam‐readcount (ver. 0.8.0) (https://github.com/genome/bam‐readcount). These parameters and cut‐off values were determined in accordance with 25469 validated mutations.

### 2.6 Construction of a catalogue of cancer‐related genes

To focus on cancer driver genes, data on 914 genes, including (a) oncogenes and tumor suppressor genes (TSGs) and (b) genes harboring somatic pathogenic mutations were compiled. The former were obtained from COSMIC Cancer Gene Census,^16^ OncoKB Cancer Gene List^17^ and the relevant literature^18^, ^19^, ^20^; the latter were obtained by integrating genes with somatic pathogenic mutations reported in CGI,^21^ ClinVar,^22^ DoCM^23^ and OncoKB^17^ and non‐functional mutations in IARC‐TP53.^24^ Furthermore, in our analysis pipeline, 1074 cancer‐related genes were compiled from 27 resources, including cancer gene panels.

### 2.7 Mutation signature

To determine the contribution of known mutational process in individual tumor samples, deconstrucSigs^25^ was used to decompose the list of mutations into 30 mutation signatures registered in COSMIC (version 2).^26^ WES‐based somatic mutations were subjected to this analysis.

### 2.8 Estimation of the tumor content

FACETS^27^ and Sequenza^28^ were used to estimate the tumor content from the results of WES. Mapping results (BAM files) for tumors and matched normal samples were supplied to these programs and the mean was used to estimate the tumor content. Except for when clearly specified, no cut‐off was applied for sample selection on the basis of the estimated tumor content.

### 2.9 Gene expression profiling using a DNA microarray analysis

We performed gene expression profiling (GEP) as described in a previous study.^29^, ^30^ Briefly, 100 ng of total RNA was amplified and subjected to fluorescent labeling. Fluorescently labeled samples were then hybridized to a SurePrint G3 Human Gene Expression 8 × 60 K v2 Microarray (Agilent Technologies). The data were analyzed with the GeneSpring GX software program (Agilent Technologies). Raw signal intensity values were log‐transformed and normalized to 75th percentile values. A signature analysis—based on the gene expression—was performed using the gene expression of the tumor and corresponding normal tissue specimens. We calculated the expression signature/score from the average of genes in the gene sets for each of the individual signatures. The signature genes for the cytolytic activity,^31^ CD8 T cells, NK cells, and B cells^32^ were retrieved from a previous study. As the signature gene sets for interferon responses and Wnt/b‐catenin signaling, we downloaded “HALLMARK INTERFERON ALPHA RESPONSE,” “GO CELLULAR RESPONSE TO INTERFERON GAMMA,” and “CHIANG LIVER CANCER SUBCLASS CTNNB1 UP” from the Molecular Signatures Database (MSigDB, version 6.1).^33^ TP53 inactivation,^34^ CIN,^35^, ^36^, ^37^ PI3K/mTOR CMAP UP,^38^, ^39^, ^40^ and T cell‐inflamed GEP^41^, ^42^, ^43^ were retrieved from a previous study. The genes of these signatures and matched sources^31^, ^32^, ^33^, ^34^, ^35^, ^36^, ^37^, ^38^, ^39^, ^40^, ^41^, ^42^, ^43^ are listed in Table S2. Ward's linkage method was applied for the hierarchical clustering of samples.

### 2.10 t‐SNE analysis

For the t‐Distributed Stochastic Neighbor Embedding (t‐SNE) analysis, we performed analyses in the “Rtsne” package (https://github.com/jkrij the/Rtsne) using our GEP dataset from the JCGA.^44^

### 2.11 Determination of CNVs

Somatic CNVs were determined using saasCNV.^45^ This method accounts for both the read depth ratio and the B allele frequency, and achieved the best performance among six CNV detection tools.^46^ All WES‐based SNVs detected via TVC in blood and tumor samples were utilized in this analysis. SNVs with a variant allele frequency of ≥90% were considered homozygous; the remaining SNVs were considered heterozygous. Three types of CNVs were extracted in accordance with the following criteria:

- Gain: *p*‐value <0.001, copy number ≥2.5
- Loss: *p*‐value <0.001, copy number ≤1.5
- Copy neutral LOH: *p*‐value <0.001, copy number = 1.5–2.5, log2.mBAF.mean.adj ≥0.2.

**Figure 1:**
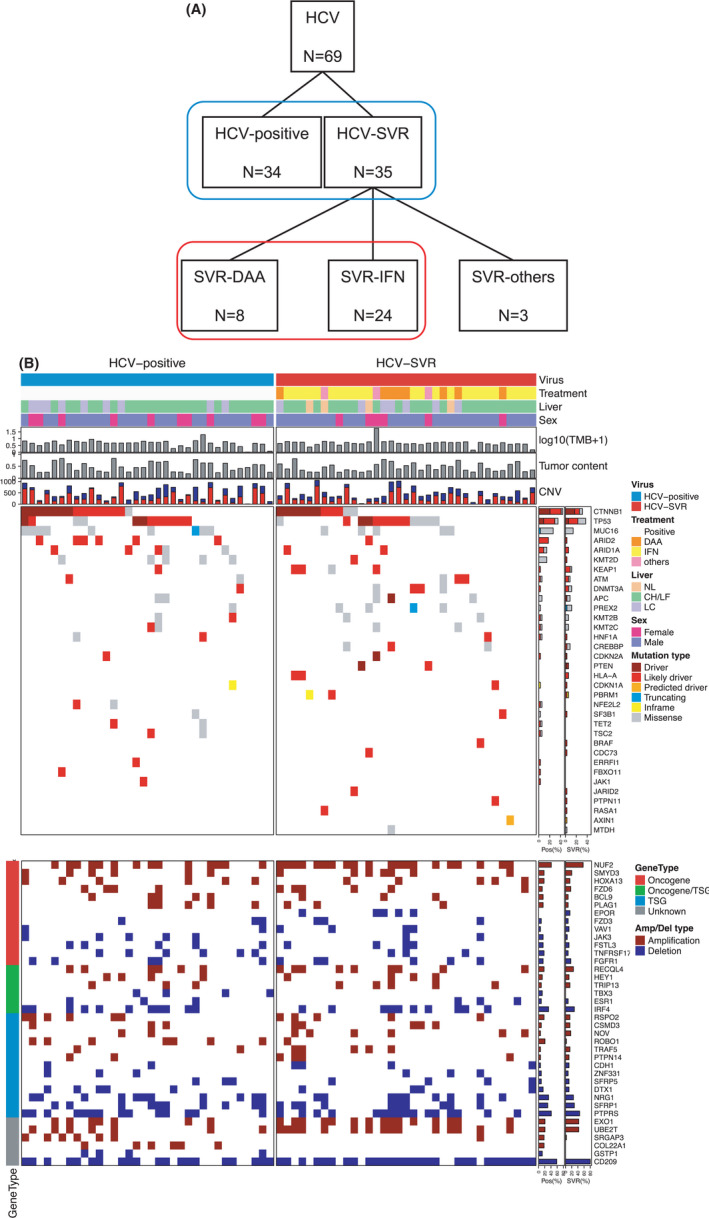
Comparison of the genomic landscape between HCV‐positive and HCV‐SVR tumors. (A) study population. (B) The top panel shows the hepatitis C virus (HCV) infection status, treatments for HCV, non‐tumorous liver conditions, sex, individual tumor mutation burden, and tumor content. The middle panel shows genes with driver mutations and the mutation types are indicated in the legend. The bottom panel displays oncogene amplification and tumor suppressor gene (TSG) deletion, which was determined via an integrative analysis of GEP and copy number variations (CNVs)

To detect driver expression aberration, an integrative analysis of GEP and CNVs was performed. Amplification (oncogene) was defined as a ≥fivefold increase and copy number ≥2.5. Deletion (TSG) was defined as a ≥ fivefold decrease and copy number ≤1.5. We added a new Figure showing the results of the analysis as an Oncoprint (Figure 1B). For all cancer‐related genes, the ratio of amplification and deletion in the two groups was compared by Fisher's exact test, and genes with *p* values of <0.1 were extracted. In addition, genes were extracted for which amplification or deletion was detected in 6 or more of the total cases of oncogene and TSG.

### 2.12 Clinical and pathological characteristics

Clinical and pathological data were collected from an HCC database that was prospectively maintained by Shizuoka Cancer Center Hospital. The tumor size was measured at the largest diameter. Pathological staging was performed according to the International Union Against Cancer tumor lymph node metastasis classification.^47^

### 2.13 Histology and immunohistochemistry

In all cases, resected specimens were fixed in 10% formalin, dehydrated, and then embedded in paraffin. The immunohistochemical analysis was performed with the use of a Bond III automated stainer and a BOND Polymer Refine Detection Kit (Leica Biosystems). The sections were reacted with anti‐P53 (DO‐7) mouse monoclonal antibody at 1:100 dilution (Dako). For the assessment of the p53 expression, the percentage of the total cell population that expressed p53 was evaluated in the tumor and in the non‐tumorous liver for each case. A sample was classified as p53 mutation‐positive if ≥30% of cancer cells showed a distinct nuclear immunoreaction. A sample was classified as null‐type if an immunoreaction was not observed. The remaining cases were judged as wild‐type.

### 2.14 Statistical analysis

The Mann–Whitney *U* test was used for the comparison of continuous variables, which were expressed as the median and interquantile range. A chi‐squared test and Fisher's exact probability test were performed for the univariate analysis of categorical variables. Spearman's correlation test was used to assess correlation. The Kaplan–Meier method was used for the evaluation of overall survival (OS) and relapse‐free survival (RFS). The statistical significance of differences was calculated using a log‐rank test. The JMP software package (version 14.0 for Mac; SAS Institute Inc.) was used to perform all statistical analyses. *p* values of <0.05 were considered to indicate statistical significance.

## 3 RESULTS

### 3.1 Clinicopathological factors in eligible patients

Among 69 HCCs with HCV, 35 HCCs were classified as HCV‐SVR, and 34 HCCs were classified as HCV‐positive. Of the 34 HCV‐positive patients, 11 had been treated with IFN, and 3 had been treated with DAAs. The characteristics and clinicopathological factors of the patients are shown in Table 1. The ICG R _15_ value was lower (*p* = 0.001) and the tumor size was smaller (*p* = 0.002) in HCV‐SVR tumors than in HCV‐positive tumors. The tumor stage was more advanced in HCV‐positive tumors than in HCV‐SVR tumors (*p* = 0.011). In two patients, hepatitis C resolved itself without specific treatment, and one patient received only ribavirin and achieved an SVR. These samples were classified as SVR‐others and were excluded from the analyses that compared treatment for HCV (Figure 1A). The median follow‐up period was 43.8 months and the 5‐year OS rate was 86.3%. No significant difference was observed in the OS or RFS rates of HCV‐SVR and HCV‐positive patients (Figure S1).

**Table 1:** Clinicopathological factors according to HCV treatment

Variable		All patients	HCV‐positive	HCV‐SVR	*p*‐value^b^
		*N* = 69	*N* = 34	*N* = 35	
Patients' characteristics					
Sex, *N* (%)	Male	53 (77%)	24 (71%)	29 (83%)	0.226
	Female	16 (23%)	10 (29%)	6 (17%)	
Age, years old, (IQR)^a^		70 (66–76)	72 (66–78)	68 (64–75)	0.241
ICG‐R_15_, %, (IQR)^a^		11.2 (7.9–14.6)	12.7 (10.8–16.4)	9.0 (6.8–11.3)	**0.001** ^c^
AFP, ng/ml, (IQR)^a^		13.2 (4.6–111.3)	20.5 (3.5–132.7)	9.2 (5.5–32.3)	0.666^c^
PIVKAII, mAU/ml, (IQR)^a^		95.0 (24.8–646.5)	114.5 (31.8–1340.3)	72.5 (20.5–435.0)	0.175^c^
Pathological factors					
Tumor size, mm, (IQR)^a^			37 (25–49)	24 (17–35)	**0.002** ^c^
Differentiation	well	13 (19%)	4 (12%)	0 (0%)	0.067
	moderate	54 (78%)	30 (88%)	24 (69%)	
	poor	2 (3%)	0 (0%)	2 (6%)	
Growth pattern	Expansive	64 (93%)	31 (91%)	33 (94%)	0.618
	Invasive	5 (7%)	3 (9%)	2 (6%)	
Fibrous capsule	positive	57 (83%)	29 (85%)	28 (80%)	0.561
Portal vein invasion	Vp0	50 (72%)	24 (71%)	26 (74%)	0.238
	Vp1	16 (23%)	8 (24%)	8 (23%)	
	Vp2	1 (1%)	0 (0%)	1 (3%)	
	Vp3	2 (3%)	2 (6%)	0 (0%)	
Hepatic vein invasion	Vv0	60 (87%)	29 (85%)	31 (89%)	0.487
	Vv1	8 (12%)	4 (12%)	4 (11%)	
	Vv2	1 (1%)	1 (3%)	0 (0%)	
T (UICC 8th)	1	53 (77%)	21 (62%)	32 (91%)	0.141
	2	11 (16%)	9 (26%)	2 (6%)	
	3	1 (1%)	0 (0%)	1 (3%)	
	4	4 (6%)	4 (12%)	0 (0%)	
N (UICC 8th)	0	69 (100%)	34 (100%)	35 (100%)	NA
	1	0 (0%)	0 (0%)	0 (0%)	
M (UICC 8th)	0	69 (100%)	34 (100%)	35 (100%)	NA
	1	0 (0%)	0 (0%)	0 (0%)	
Stage (UICC 8th)	I	53 (77%)	21 (62%)	32 (91%)	**0.011**
	II	11 (16%)	9 (26%)	2 (6%)	
	III	5 (7%)	4 (12%)	1 (3%)	
Non‐tumorous liver	NL	4 (6%)	0 (0%)	4 (11%)	0.055
	CH/LF	49 (71%)	25 (74%)	24 (69%)	
	LC	16 (23%)	9 (26%)	7 (20%)	

*Note*: Values in parentheses are percentages unless indicated otherwise. Abbreviations: AFP, alpha fetoprotein; CH, chronic hepatitis; HCV, hepatitis C virus; ICG, indocyanine green; LC, liver cirrhosis; LF, liver fibrosis; NL, normal liver; SVR, sustained virological response. The Vp grades were defined as follows: Vp0, absence of tumor thrombus; Vp1, invasion or tumor thrombus distal to the second branch of the portal vein; and Vp2, invasion or tumor thrombus in the second branch of the portal vein. The Vv grades were defined as follows: Vp0, absence of tumor thrombus; Vv1, tumor thrombus in the peripheral hepatic vein branch; and Vv2, tumor thrombus in the main trunk of the hepatic vein.

^a^ Values are the median (interquartile range).

^b^ χ^2^ test unless indicated otherwise.

^c^ Mann–Whitney *U* test. Significant values are indicated with bold typeface.

### 3.2 Driver somatic alterations according to whether HCV‐SVR was achieved

To evaluate the genomic alterations contributing to hepatocarcinogenesis, the results of WES were analyzed between HCV‐positive and HCV‐SVR tumors. The landscape of mutations according to the HCV statuses are shown in Figure 1B. Although the rate and exclusivity of driver mutations in *CTNNB1* and *TP53* in the two groups did not differ to a statistically significant extent, in cases without driver mutations in both *CTNNB1* and *TP53*, there seemed to be a tendency for different genes to detect driver mutations. A significant difference in the mutation frequency was identified between HCV‐positive and HCV‐SVR tumors. The frequency of tumors with *ARID2* mutations was lower in HCV‐SVR tumors than in HCV‐positive tumors (2.9% vs. 17.7%, *p* = 0.048). In the TCGA liver cancer cohort, the OS of cases with *ARID2* mutation and cases without ARID2 mutations did not differ to a statistically significant extent (Figure S2A). In contrast, the frequency of samples with mutations in *PREX2* in HCV‐SVR tumors was higher than that in HCV‐positive tumors (11.4% vs. 2.9%, *p* = 0.048). In the TCGA liver cancer cohort, cases with *PREX2* mutations had significantly shorter OS in comparison to cases without *PREX2* mutations (Figure S2B). The frequency of samples with mutations in *KEAP1*, which is known to promote malignant potential in HCC in HCV‐SVR tumors, was also higher (11.4%) than that in HCV‐positive tumors (2.9%). The viral status, tumor mutation burden (TMB, mutation per megabase) and mutational signatures are shown in Figure S3. A comparison of the TMB between HCV‐positive tumors and HCV‐SVR tumors identified no significant difference. Thirty mutational signatures of the COSMIC database were investigated using deconstructSigs,^25^ which confirmed that the mutational signatures of HCV‐positive tumors and HCV‐SVR tumors did not differ to a statistically significant extent.

### 3.3 Gene amplification and deletion according to the status of HCV

Copy number variations (CNVs) were estimated on the basis of the relative sequence coverage of normal tissue and tumor pairs. Oncogene amplification (≥fivefold increase and copy number ≥2.5) was assessed via an integrative analysis of GEP and CNVs (Figure 1C). A significant difference in the amplification frequency was identified between HCV‐positive and HCV‐SVR tumors. The frequency of amplifications identified in *ROBO1* (*p* = 0.024), *COL22A1* (*p* = 0.011), and *SRGAP3* (*p* = 0.048) was significantly lower in HCV‐SVR tumors than in HCV‐positive tumors. To investigate the prognostic significance of the expression of these genes in a large cohort, a survival analysis was performed using TCGA RNA‐sequencing data in the liver cancer cohort. Patients with the high expression of *ROBO1* and those with the high expression of *COL22A1* showed worse outcomes (Figure S4).

### 3.4 Total gene expression patterns according to the HCV status and treatments for HCV

A two‐dimensional t‐SNE analysis was performed using the “Rtsne” package, based on comprehensive GEP data in Figure S5. The t‐SNE plots were distributed along with the expression. There was no obvious difference in the expression pattern according to the HCV status and treatments for HCV. In addition, the evaluation of the correlation between the driver mutations and the expression pattern revealed no clear association between the expression pattern and gene mutations.

### 3.5 Alterations in the tumor microenvironment and inflammation after HCV clearance

**Figure 2:**
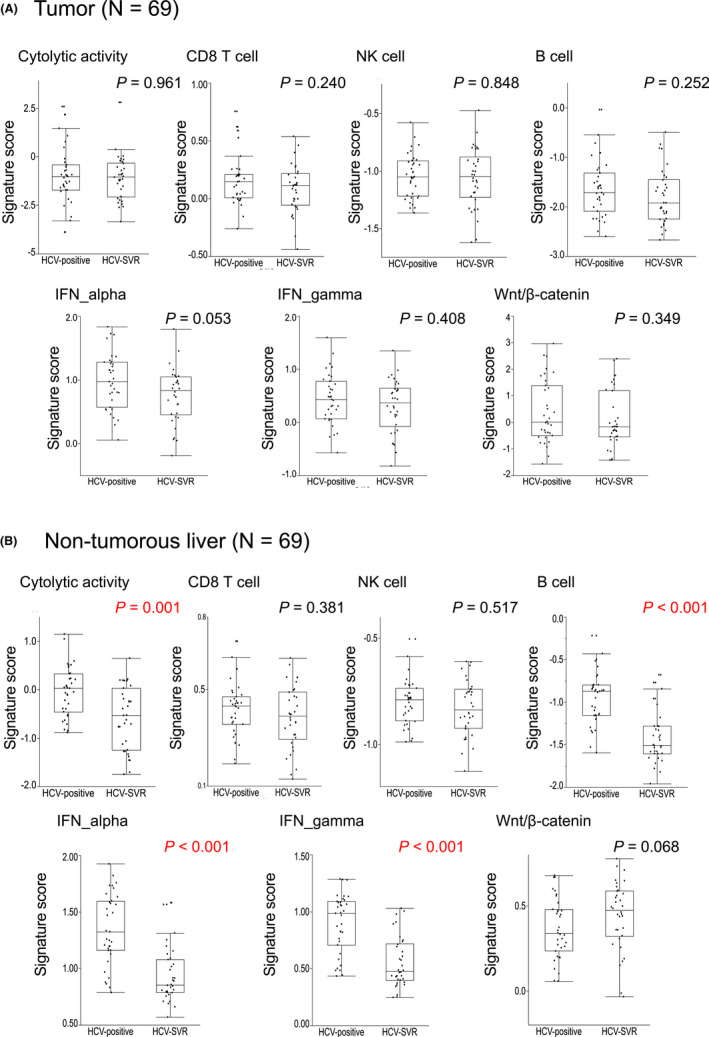
Comparison of mutational features and tumor microenvironment between HCV‐positive and HCV‐SVR tumors. (A) Comparisons of the tumor microenvironment in HCV‐positive and HCV‐SVR tumors. Significant differences in signatures associated with the tumor microenvironment were not identified. (B) Comparisons of the tumor microenvironment between HCV‐positive and HCV‐SVR non‐tumorous adjacent liver. *p < 0.05 (Mann–Whitney *U* test)

Although most HCCs arise on a background of chronic inflammation induced by HCV infection, the tumor microenvironment is immunosuppressive. However, the correlation between these mechanisms and HCV clearance in clinical samples is unclear. To investigate this issue, we performed unsupervised hierarchical clustering of HCC tumor tissues and paired adjacent non‐tumorous liver tissues using gene expression signatures. For the gene signatures associated with tumor immune and WNT/β‐catenin signaling, we used a previously defined gene set (Table S2). Figure 2A shows the results of clustering analyses and comparisons of gene expression signatures in HCV‐positive tumors and HCV‐SVR tumors. Significant differences in signatures associated with tumor microenvironment were not identified. The results of clustering analyses and comparisons of gene expression signatures in HCV‐positive tumors and HCV‐SVR non‐tumorous adjacent liver are shown in Figure 2B. Wnt/β‐catenin signaling tended to be enhanced in HCV‐SVR cases in comparison to the liver in HCV‐positive cases. Notably, the cytolytic activity (*p* = 0.001), B cell (*p* < 0.001), INF_alpha response (*p* < 0.001), and IFN_gamma response (*p* < 0.001) signatures in the non‐tumorous liver in the HCV‐SVR group were significantly more suppressed in comparison to the HCV‐positive group. Taken together, tumor inflammation was poorly correlated with HCV clearance, indicating that the tumor microenvironment is less influenced by background liver inflammation.

### 3.6 Genetic alterations according to HCV treatment

**Figure 3:**
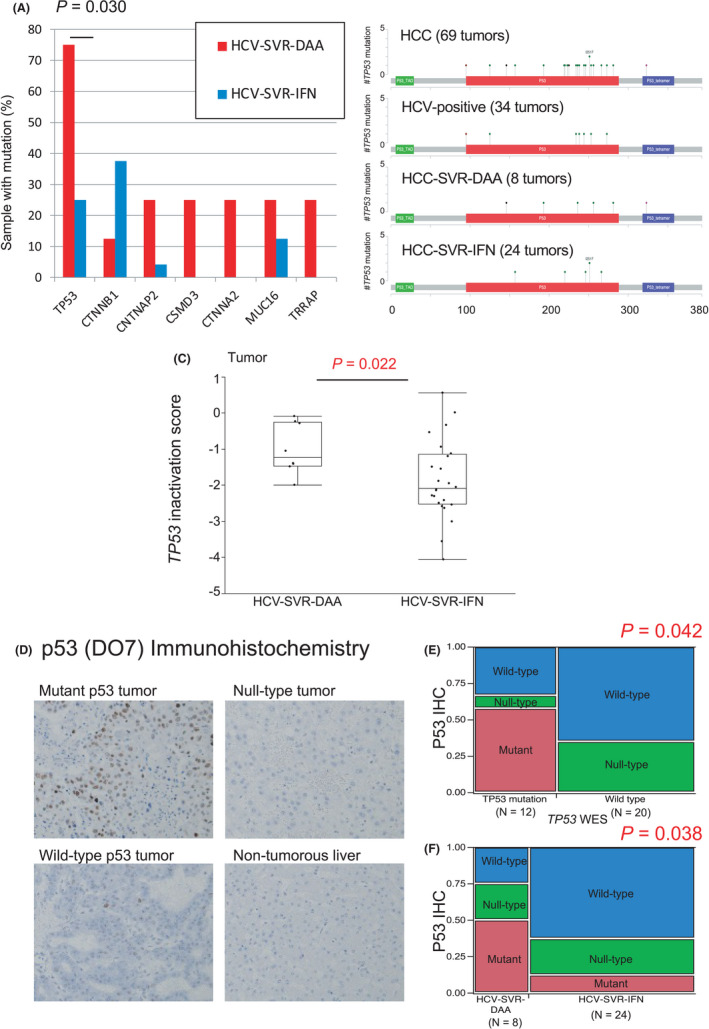
Genetic alterations in HCC after HCV‐SVR according to treatment for HCV. (a) Frequency of driver alterations according to treatment for HCV. *p < 0.05 (chi‐squared test). (B) Mutation mapping in *TP53* according to HCV status. (C) Comparison of *TP53* inactivation score between HCV‐positive and HCV‐SVR tumors. *p < 0.05 (Mann–Whitney *U* test) (D) Representative image of immunohistochemistry for mutant p53, null‐type, wild‐type, and non‐tumorous liver. (E) Combined mutant and null‐type p53 were relatively in accordance with *TP53* mutations (*p* = 0.042) (F) Frequency of samples with mutant p53 (mutant and null‐type) was significantly higher in HCV‐SVR‐DAA than in HCV‐SVR‐IFN tumors (*p* = 0.038)

To investigate the effect of SVR treatment on genomic alterations, we next compared somatic mutations identified in eight HCV‐SVR‐DAA tumors and 24 HCV‐SVR‐IFN tumors. The frequency of mutations according to HCV treatment is shown in Figure 3A. The frequency of samples with mutations in *TP53* (*p* = 0.030) was significantly higher in HCV‐SVR‐DAA than in HCV‐SVR‐IFN tumors. There was no significant difference in the *ARID2* (0.0% vs. 4.2%, *p* = 0.444) or *PREX2* (12.5% vs. 12.5%, *p* = 1.000) mutation rates between HCV‐SVR‐DAA and HCV‐SVR‐IFN tumors. Mutational positions in *TP53* were not localized to specific regions regardless of the treatment for HCV (Figure 3B). Furthermore, based on previous expression analyses,^34^, ^48^ we used the expression levels of four genes (*CDC20*, *PLK1*, *CENPA*, and *KIF2C*) to calculate the *TP53* inactivation score. This *TP53* inactivation score was significantly more enhanced in HCV‐SVR‐DAA tumors than in HCV‐SVR‐IFN tumors (*p* = 0.022, Figure 3C). To validate the results at the protein level, we performed immunohistochemical staining using an anti‐p53 antibody. Representative images of mutant p53, null‐type, and wild‐type are shown in Figure 3D. The p53 expression was negative in the non‐tumorous liver cell population. Combined mutant and null‐type p53 were relatively in accordance with *TP53* mutations (*p* = 0.042, Figure 3E), and the frequency of samples with combined mutant and null‐type p53 was significantly higher in HCV‐SVR‐DAA than in HCV‐SVR‐IFN tumors (*p* = 0.038, Figure 3F).

### 3.7 Influence of TP53 inactivation on chromosomal instability and the PI3K/AKT/mTOR pathway

**Figure 4:**
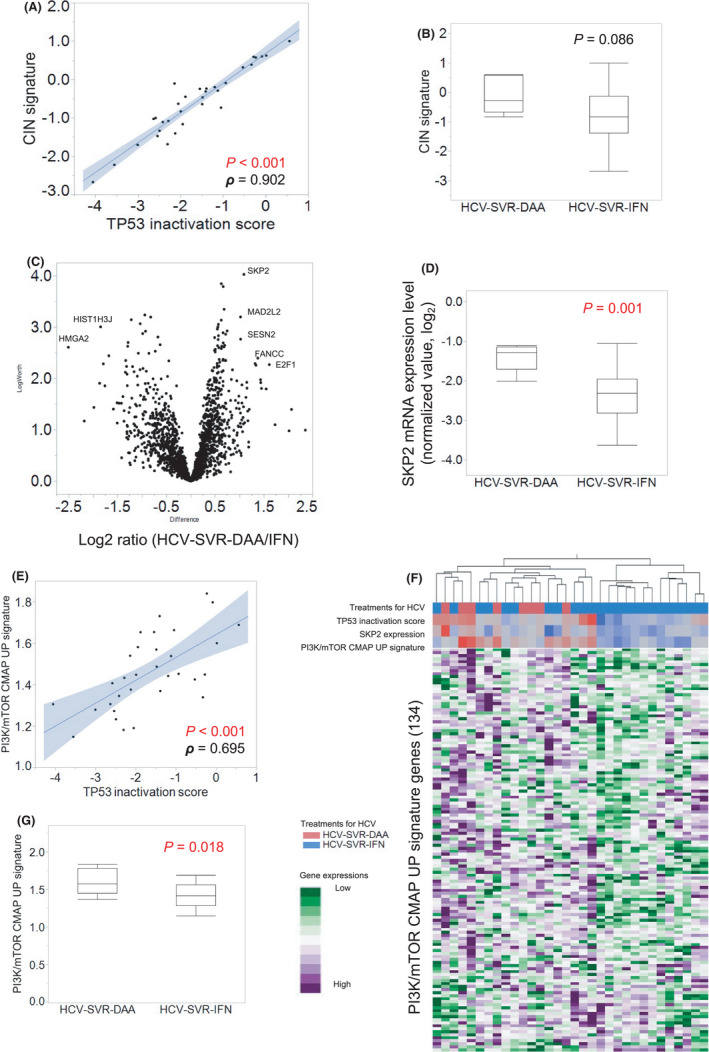
Influence of *TP53* inactivation on chromosomal instability and the PI3K/AKT/mTOR pathway. (A) The *TP53* inactivation score was highly correlated with the CIN signature (*p* < 0.001, rho value = 0.902, Spearman's correlation). (B) The CIN signature was increased in HCV‐SVR‐DAA tumors compared with HCV‐SVR‐IFN tumors. (C) Volcano plot showing the results of microarray analysis in HCV‐SVR‐DAA tumors and HCV‐SVR‐IFN tumors. (D) *SKP2* was identified as being especially overexpressed in HCV‐SVR‐DAA tumors. (E) Analysis of the correlation between the PI3K/mTOR CMAP UP signature and the *TP53* inactivation score showed a significant positive correlation (*p* < 0.001, rho value = 0.695, Spearman's correlation). (F) Clustering analysis of the expression of 134 genes in the PI3K/mTOR CMAP UP signature. (g) The PI3K/mTOR CMAP UP signature was significantly enhanced in HCV‐SVR‐DAA tumors (*p* = 0.018)

Chromosomal instability (CIN) is known to occur in association with a defect of the *TP53* function.^34^ To test the correlation between TP53 inactivation and CIN in HCC‐SVR tumors, a correlation analysis was performed using the gene expression score and signatures. A strong correlation was observed between the *TP53* inactivation score and the CIN signature (*p* < 0.001, rho value = 0.902, Spearman's correlation test, Figure 4A). This CIN signature was increased in HCV‐SVR‐DAA tumors (Figure 4B). To validate the findings for CIN, we performed a CNV analysis (Figure S6). The CNV map is shown in Figure S5A, which demonstrates no significant difference in the accumulation of CNVs between HCV‐SVR‐DAA and HCV‐SVR‐IFN. To further investigate of the difference of CNVs according to the HCV treatment, CNV was classified into amplification (Figure S5B) and deletion (Figure S5C). The analysis revealed that deletion was more frequently identified in HCV‐SVR‐DAA tumors than in HCV‐SVR‐IFN tumors (*p* = 0.016). Based on these results, it is considered that *TP53* activation—which is related to both CIN and CNV—was suppressed in tumors of the HCV‐SVR‐DAA group. We performed a volcano plot analysis for GEP in order to determine variations in the expression of other tumorigenesis‐related genes (Figure 4C). *SKP2* was identified as being especially overexpressed in HCV‐SVR‐DAA tumors in comparison to HCV‐SVR‐IFN tumors (*p* < 0.001, Figure 4D), and is known to be correlated with the PI3K/AKT/mTOR pathway.^49^, ^50^ The correlation between the PI3K/mTOR CMAP UP signature and the *TP53* inactivation score was analyzed to investigate the correlation between *TP53* inactivation and the PI3K/AKT/mTOR pathway. This analysis revealed a significant positive correlation (*p* < 0.001, rho value = 0.695, Spearman's correlation test, Figure 4E). A clustering analysis of the expression of the 134 genes in the PI3K/mTOR CMAP UP signature^38^, ^40^ showed that most of these genes had a moderately positive correlation with *TP53* inactivation, and upregulation of these genes was evident in HCV‐SVR‐DAA tumors accompanied by the upregulation of *SKP2* (Figure 4F). The PI3K/mTOR CMAP UP signature was significantly enhanced in HCV‐SVR‐DAA tumors (*p* = 0.018, Figure 4G).

### 3.8 Comparison of clinical and pathological features and the outcomes of HCV‐SVR‐DAA and IFN

Table 2 shows the patient characteristics and pathological findings of the resected specimens. Although no significant difference was observed in tumor markers, differentiation, or the TNM stage between HCV‐SVR‐DAA and HCV‐SVR‐IFN, HCV‐SVR‐DAA was significantly associated with portal vein invasion (*p* = 0.023) in comparison to HCV‐SVR‐IFN. Regarding the pathological findings of the non‐tumorous liver, liver cirrhosis was significantly more frequently observed in HCV‐SVR‐DAA than in HCV‐SVR‐IFN (*p* = 0.006), indicating that HCC can occur on a background of severe fibrotic liver after HCV‐SVR by DAA, and that HCC can occur on a normal liver background after HCV‐SVR by IFN. A comparison of the clinical features according to the *TP53* mutation status in HCV‐SVR tumors is shown in Table S2. Liver cirrhosis was also significantly more frequently observed in tumors with *TP53* mutation than in tumors with wild type *TP53* (*P* = 0.010). No significant difference was observed in OS or RFS between HCV‐SVR‐DAA and HCV‐SVR‐IFN in the present cohort, although cases with TP53 mutation had significantly shorter OS in comparison to cases without TP53 mutations in the TCGA liver cancer cohort (Figure S7).

**Table 2:** Clinicopathological factors according to HCV treatment

Variable		HCV‐SVR		*p*‐value^b^
		DAA	IFN	
		*N* = 8	*N*=24	
Patients' characteristics				
Sex, *N* (%)	Male	6 (75%)	22 (92%)	0.246
	Female	2 (25%)	2 (8%)	
Age, years old, (IQR)		68 (66–77)^a^	69 (61–74)^a^	0.983^c^
ICG‐R15, %, (IQR)		10 (6–14)^a^	9 (6–10)^a^	0.384^c^
AFP, ng/ml, (IQR)		12 (6–28)^a^	9 (6–96)^a^	0.931^c^
PIVKAII, mAU/ml, (IQR)		165 (61–654)^a^	55 (19–431)^a^	0.240^c^
Pathological factors				
Tumor size, mm, (IQR)		18 (12–26)^a^	24 (19–35)^a^	0.127^c^
Differentiation	well	4 (50%)	5 (21%)	0.251
	moderate	4 (50%)	18 (75%)	
	poor	0 (0%)	1 (4%)	
Growth pattern	Expansive	7 (88%)	23 (96%)	0.431
	Invasive	1 (13%)	1 (4%)	
Fibrous capsule	positive	6 (75%)	19 (79%)	0.807
Portal vein invasion	Vp0	3 (38%)	20 (83%)	**0.023**
	Vp1	5 (63%)	3 (13%)	
	Vp2	0 (0%)	1 (4%)	
Hepatic vein invasion	Vv0	8 (100%)	20 (83%)	0.115
	Vv1	0 (0%)	4 (17%)	
	Vv2	0 (0%)	0 (0%)	
T (UICC 8th)	1	7 (88%)	22 (92%)	0.559
	2	1 (13%)	1 (4%)	
	3	0 (0%)	1 (4%)	
	4	0 (0%)	0 (0%)	
N (UICC 8th)	0	8 (100%)	24 (100%)	N.A
	1	0 (0%)	0 (0%)	
M (UICC 8th)	0	8 (100%)	24 (100%)	N.A
	1	0 (0%)	0 (0%)	
Stage (UICC 8th)	I	7 (88%)	22 (92%)	0.559
	II	1 (13%)	1 (4%)	
	III	0 (0%)	1 (4%)	
Non‐tumorous liver	NL	0 (0%)	3 (13%)	**0.006**
	CH/LF	3 (38%)	19 (79%)	
	LC	5 (63%)	2 (8%)	

*Notes*: Values in parentheses are percentages unless indicated otherwise. Abbreviations: AFP, alpha fetoprotein; CH, chronic hepatitis; HCV, hepatitis C virus; ICG, indocyanine green; LC, liver cirrhosis; LF, liver fibrosis; NL, normal liver; SVR, sustained virological response. The Vp grades were defined as follows: Vp0, absence of tumor thrombus; Vp1, invasion or tumor thrombus distal to the second branch of the portal vein; and Vp2, invasion or tumor thrombus in the second branch of the portal vein. The Vv grades were defined as follows: Vp0, absence of tumor thrombus; Vv1, tumor thrombus in the peripheral hepatic vein branch; and Vv2, tumor thrombus in the main trunk of the hepatic vein.

^a^ Values are the median (interquartile range).

^b^ χ^2^ test unless indicated otherwise.

^c^ Mann–Whitney *U* test.

Significant values are indicated with bold typeface.

## 4 DISCUSSION

In the present study, our comprehensive genetic profiling by WES revealed significant differences in the mutation rate of several driver genes between HCV‐positive and HCV‐SVR tumors. Mutations in *ARID2* were significantly less frequent in HCV‐SVR tumors than in HCV‐positive tumors. Mutations of the *ARID2* gene, which is involved in chromatin remodeling gene, are found in many human cancers.^51^, ^52^ In HCC, *ARID2* mutations are reported to occur more frequently in HCV‐induced HCCs (6/43) in comparison to HBV‐related (1/50) and non‐viral‐related (2/44) HCC.^53^ In our results, *ARID2* mutations were observed in HCV‐positive tumors (6/34) and in HCV‐SVR tumors (1/35). These findings indicated that HCV clearance might prevent mutations in *ARID2* caused by HCV infection, suggesting that *ARID2* is a potential critical molecule for hepatocarcinogenesis caused by HCV infection. However, mutations in *PREX2* were observed at a significantly higher frequency in HCV‐SVR tumors than in HCV‐positive tumors. The overexpression of *PREX2* has been reported in various tumors, including HCC.^54^ According to our analysis of data obtained from public‐domain databases, the mutation rate of *PREX2* in HCC was 6.4%–28.3%. A gain‐of‐function mutation in *PREX2* in HCC was previously reported to promote cell migration.^55^ *KEAP1* was also identified as a gene with a higher frequency of mutation in HCV‐SVR tumors. Inactivating mutations of *KEAP1* are commonly reported in HCCs.^56^, ^57^ These alterations result in the accumulation of NRF2 protein and confer resistance to oxidative stress and induce metabolic transformation in cancer cells.^58^ Thus, the higher mutation rates of *PREX2* and *KEAP1* in HCV‐SVR tumors might indicate that these alterations have potential as a novel and specific therapeutic target and a specific biomarker for HCV‐SVR tumors.

Chronic HCV infection‐driven inflammation contributes to hepatocarcinogenesis and progressive liver fibrosis with the formation of the carcinogenic microenvironment.^7^, ^8^ However, the correlation between these mechanisms and HCV eradication in clinical samples is unclear. We found that the tumors had no significant differences in signatures according to HCV status, indicating that tumor inflammation and the tumor microenvironment are poorly correlated with HCV clearance and that the tumor microenvironment is less influenced by background liver inflammation. In contrast to tumors, the cytolytic activity in non‐tumorous liver in HCV‐SVR was less inflamed than that in HCV‐positive. The signatures of B cells and IFN responses also had significantly decreased expression levels in non‐tumorous liver in HCV‐SVR compared with that in HCV‐positive. These facts suggest that HCV eradication suppresses immunological reactions that occur in patients with chronic HCV infection and may contribute to reducing the risk of hepatocarcinogenesis. Consequently, the involvement of the microenvironment in hepatocarcinogenesis is improved by HCV‐SVR; however, when HCC occurs even after SVR, the tumor microenvironment in HCV‐SVR is comparable to that in HCV‐positive.

The finding that the mutation rate of some driver genes varied between HCV‐positive and HCV‐SVR tumors led us to investigate the differences according to HCV treatment. Genetic profiling revealed the frequency of samples with mutations in *TP53* was significantly higher in HCV‐SVR‐DAA tumors than in HCV‐SVR‐IFN tumors. Indeed, the *TP53* inactivation score was validated to be significantly enhanced in HCV‐SVR‐DAA tumors compared with that in HCV‐SVR‐IFN tumors. This result indicated that *TP53* mutation could be a biomarker for hepatocarcinogenesis after SVR by DAA and we considered that the altered *TP53* mutation rate might characterize the features of HCV‐SVR‐DAA tumors. *TP53* inactivation is associated with enhanced CIN,^34^, ^59^, ^60^, ^61^, ^62^ and a recent pan‐cancer analysis suggested that copy number changes attributed to CIN are delayed events.^63^ Therefore, HCV‐SVR‐DAA tumors may be prone to accumulating CNV due to suppressed *TP53* activation. A recent comprehensive molecular evaluation of HCC defined three major subtypes. Of these, a subtype called MS1 that harbors *TP53* mutations displayed chromosomal instability and had a significant correlation with high serum AFP levels, aggressive vascular invasion, and an unfavorable prognosis.^64^ The present results demonstrated that HCV‐SVR‐DAA tumors harbored *TP53* mutations and displayed chromosomal instability, revealing that these characteristics might match some of the features of MS1. Although the present study could not identify different prognostic outcomes and tumor markers in HCV‐SVR‐DAA tumors because of the lack of statistical power resulting from the small sample number, aggressive vascular invasion was demonstrated in HCV‐SVR‐DAA tumors. Therefore, we consider that HCV‐SVR‐DAA tumors likely belong to the MS1 subtype of HCC.

To further investigate additional features of HCV‐SVR‐DAA tumors, we searched for genes with altered expression in HCV‐SVR‐DAA tumors. Volcano plots identified *SKP2* as an upregulated gene, which is known to be correlated with the PI3K/AKT/mTOR pathway.^49^, ^50^ In HCV‐SVR‐DAA tumors, we observed that the PI3K/mTOR CMAP UP signature was significantly enhanced, and this enhancement showed a positive correlation with *TP53* inactivation. Accordingly, we hypothesize that—in HCV‐SVR‐DAA tumors—the PI3K/AKT/mTOR pathway was enhanced along with the inactivation of *TP53*. Upregulation of the PI3K/AKT/mTOR pathway, which regulates multiple cellular functions (e.g., tumorigenesis, proliferation, differentiation, and apoptosis), has been demonstrated in many cancers. A previous study of 528 HCCs evaluated by immunohistochemistry demonstrated that enhanced PI3K/AKT/mTOR signaling was correlated with poor tumor differentiation, higher TNM stage, and vascular invasion.^65^ A tissue microarray‐based study, in which 200 HCCs were analyzed,^66^ reported that the overexpression of p‐AKT and p‐mTOR was associated with the tumor grade, as well as intrahepatic metastasis, vascular invasion, the TNM stage, and a high Ki‐67 labeling index. These findings also support the clinicopathological features and characterize the HCV‐SVR‐DAA tumors. Thus, we concluded that HCV‐SVR‐DAA tumors with *TP53* mutations display chromosomal instability and enhanced PI3K/AKT/mTOR signaling, and have a significant correlation with aggressive vascular invasion.

This study was associated with several limitations. First, the number of patients in each subgroup was relatively small, especially that of HCV‐SVR‐DAAs. In more recent years, the number of resected cases of HCV‐SVR‐DAAs has increased, and further accumulation of evidence is warranted. Second, the postoperative follow‐up period was relatively short. Accordingly, larger further studies should be performed with study populations and longer follow‐up periods in order to validate the results of the present study and investigate the mid‐term and long‐term postoperative outcomes. Third, *TERT* promoter mutations are reported to have been found in 54% of human HCCs and 25% of cirrhotic preneoplastic nodules.^67^ Taken together, this alteration may be the earliest recurrent genetic event to occur in the process of hepatocarcinogenesis. However, the present study could not investigate the most common alteration because of the lack of sequencing at the promoter region.

Our dataset potentially serves as a fundamental resource for the optimal management of HCV‐SVR‐DAA patients. Our comprehensive genetic profiling by WES revealed significant differences in the mutation rates of several driver genes between HCV‐positive and HCV‐SVR tumors. Furthermore, the frequency of samples with mutations in *TP53* was found to be significantly higher in HCV‐SVR‐DAA tumors than in HCV‐SVR‐IFN. These findings may accelerate individualized patient management in the future.

### STATEMENT OF ETHICS

The study was designed according to the Ethical Guidelines for Human Genome and Genetic Analysis Research revised in 2013. Written consent was obtained from all patients participating in the study. The present study was approved by the institutional review board of Shizuoka Cancer Center (approval no. 25–33, and 202–22‐2020‐1‐2). The study protocol conforms to the ethical guidelines of the Declaration of Helsinki.

## CONFLICT OF INTEREST

The authors declare no conflict of interest in association with the present study.

## AUTHOR CONTRIBUTIONS

Study concept and design: TI, YO, and KO; Acquisition of data: KU, TS, YO, YK, TI, YY, RA, KO, SO, TS, and TI**;** Analysis and interpretation of data: TI, YO, TN, KU, and KY**;** Drafting of the manuscript: TI, YO, and KO**;** Critical revision of manuscript: YO, KO, TS, KH, KU, YA, and KY; Statistical analysis: TI and YO**;** Study Supervision: KO and KY.

## Supporting information


Figure S1

Figure S2

Figure S3

Figure S4

Figure S5

Figure S6

Figure S7

Table S1

Table S2

## Data Availability

Data for this study is confidential patient information regulated by the IRB of the institution. Requests to access data will have to be in compliance with the institutional IRB.
