# Supplementary figures and images for: Hepatocellular carcinoma after a sustained virological response by direct‐acting antivirals harbors TP53 inactivation
Source: Cancer Med. 2022 Feb 17;11(8):1769–86. doi: 10.1002/cam4.4571 (PMC9041076; doi:10.1002/cam4.4571)

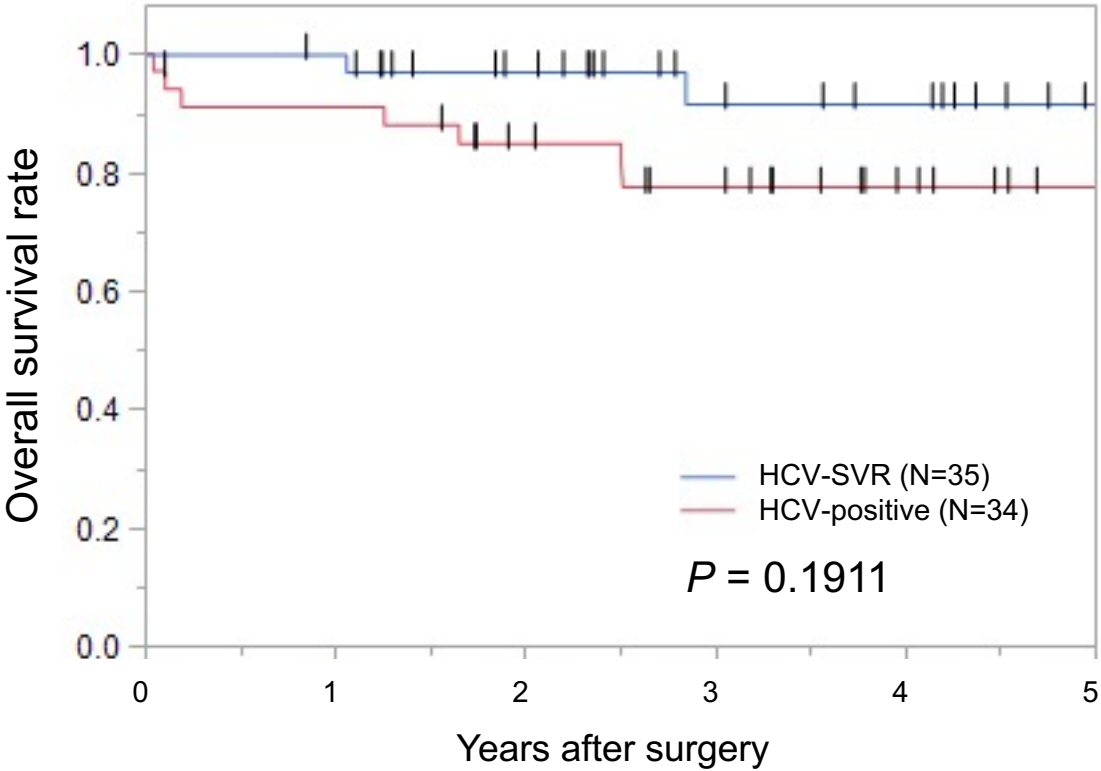

| No. at risk  |    |    |    |    |    |   |  |
|--------------|----|----|----|----|----|---|--|
| HCV-SVR      | 35 | 35 | 27 | 18 | 14 | 7 |  |
| HCV-positive | 34 | 31 | 25 | 20 | 12 | 7 |  |

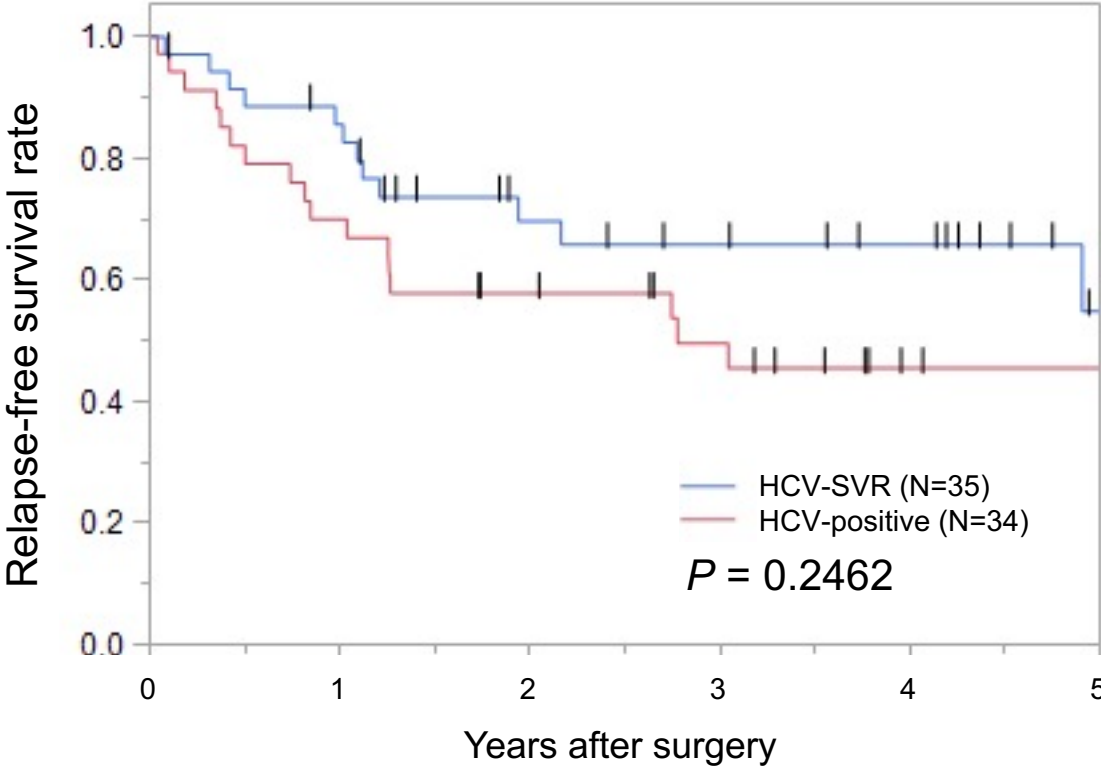

| No. at risk  |    |    |    |    |    |   |  |
|--------------|----|----|----|----|----|---|--|
| HCV-SVR      | 35 | 30 | 19 | 16 | 13 | 5 |  |
| HCV-positive | 34 | 24 | 18 | 13 | 7  | 5 |  |

Supplement: Supplementary file 1 — Figure S1 [file CAM4-11-1769-s002.pdf]

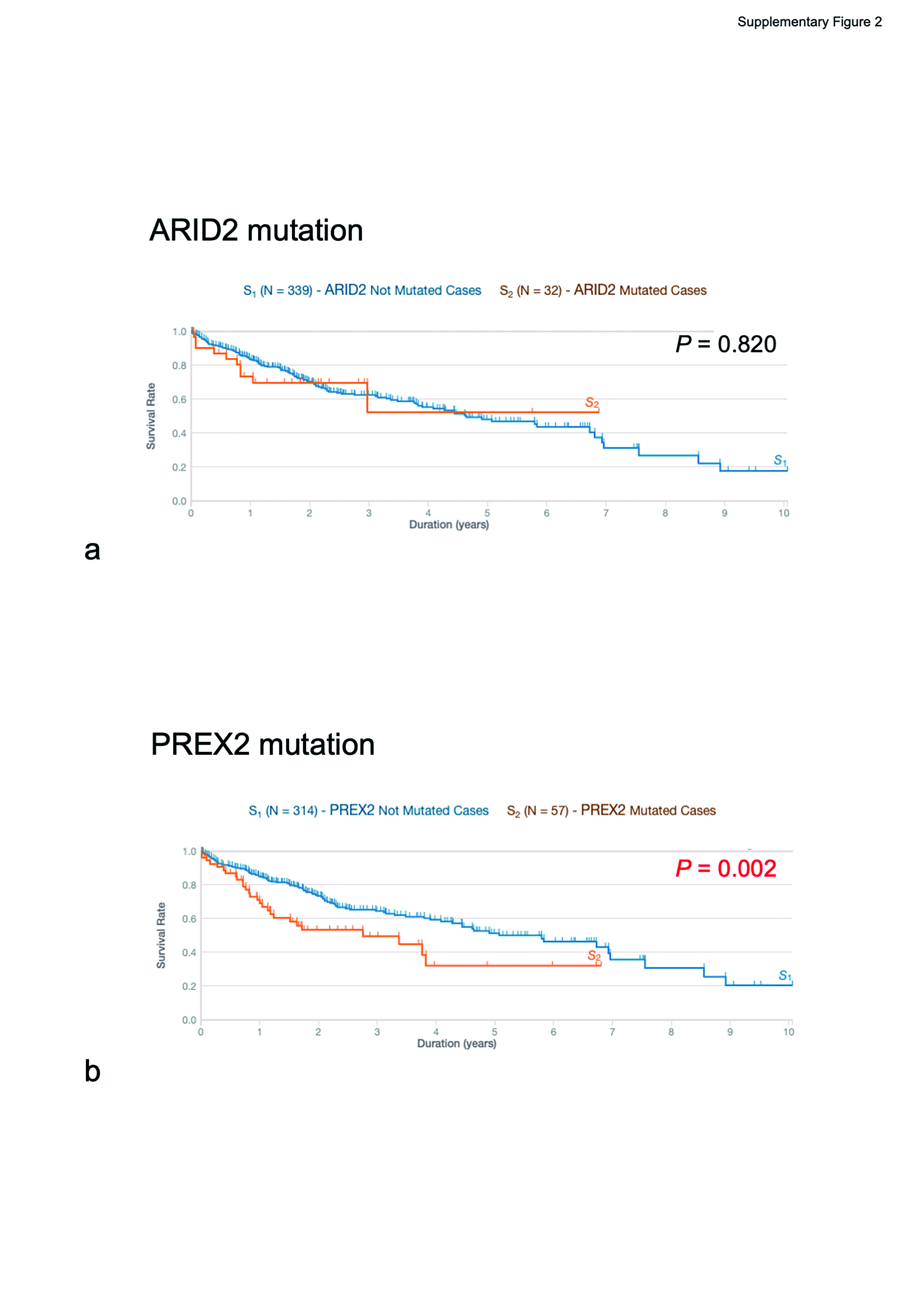

Supplement: Supplementary file 2 — Figure S2 [file CAM4-11-1769-s005.tiff]

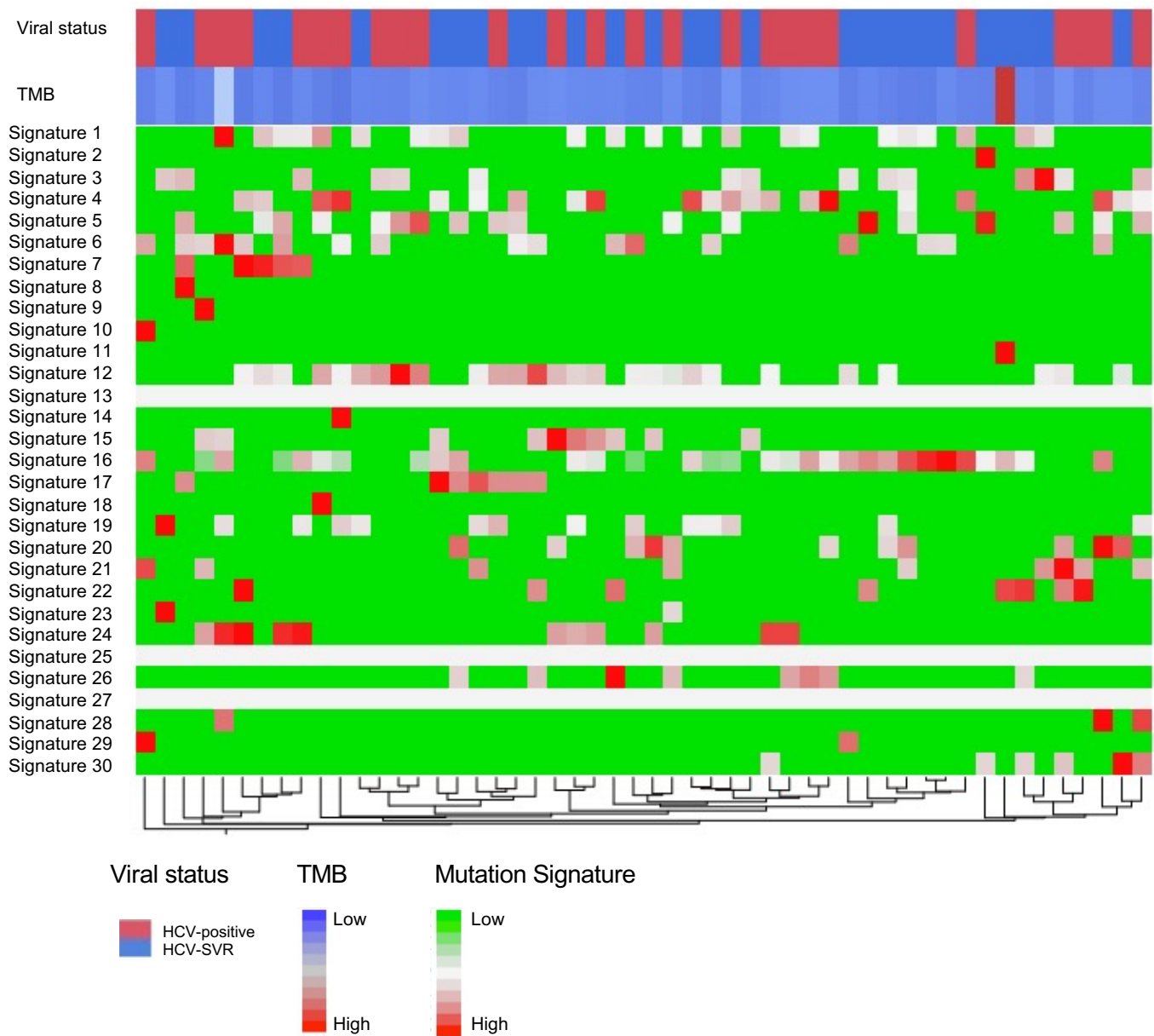

Supplement: Supplementary file 3 — Figure S3 [file CAM4-11-1769-s004.pdf]

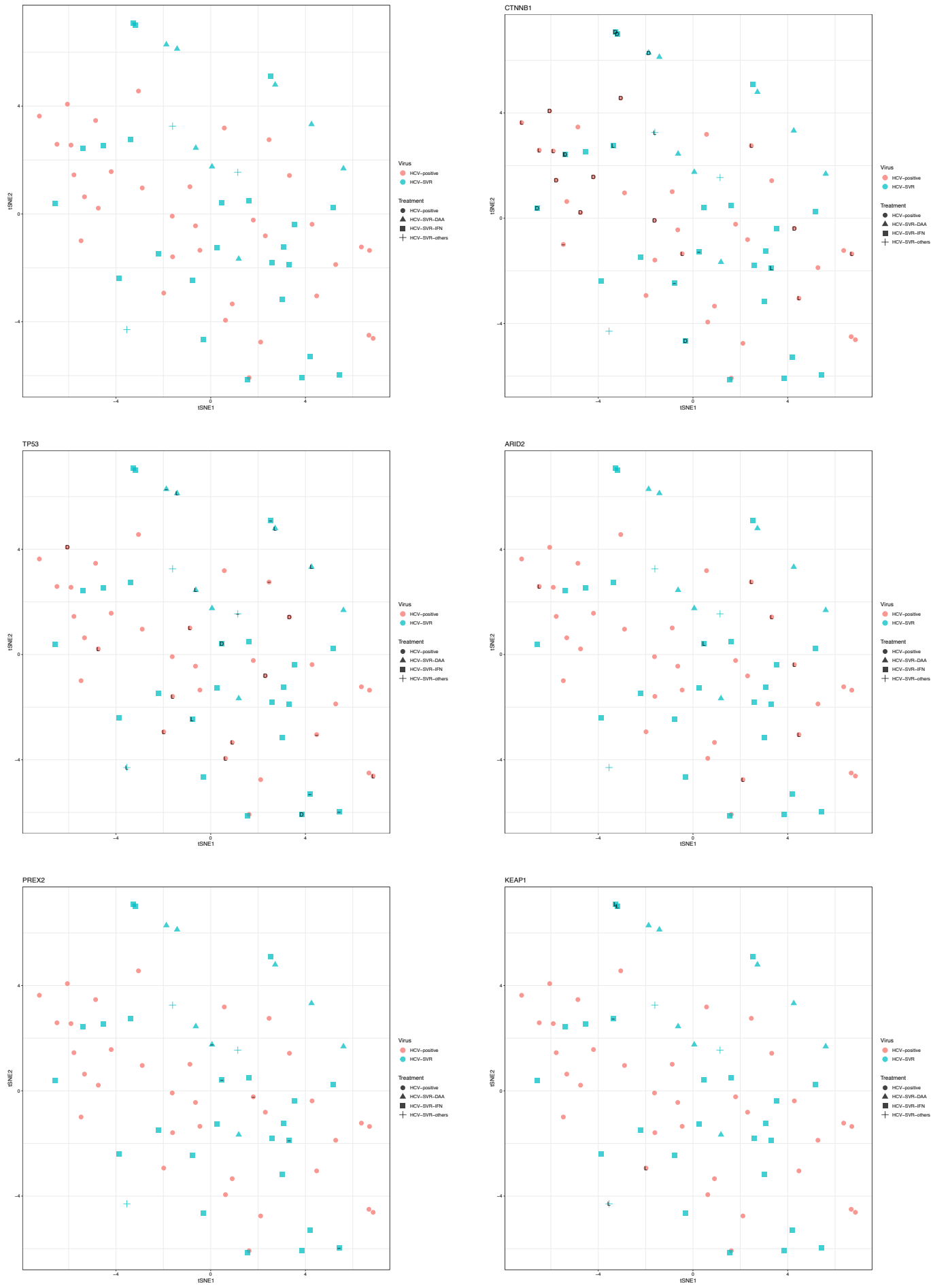

Supplement: Supplementary file 5 — Figure S5 [file CAM4-11-1769-s001.pdf]

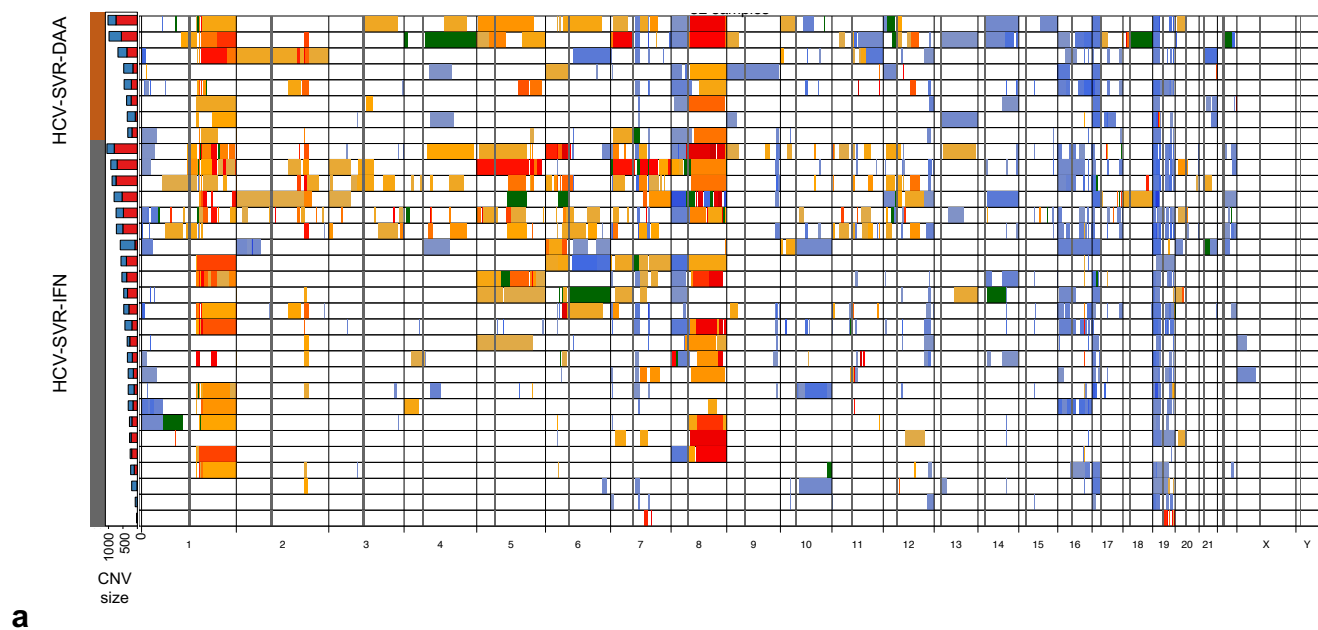**a**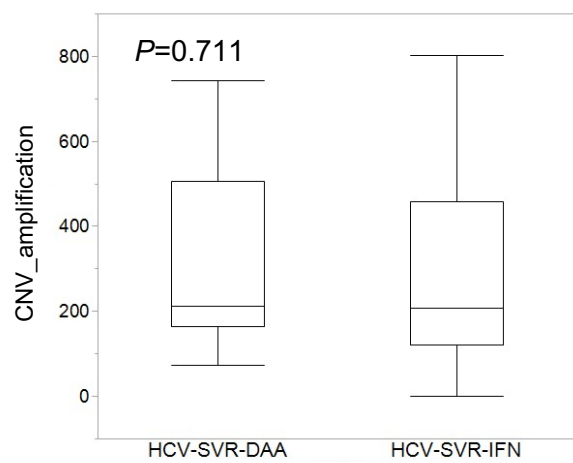**b**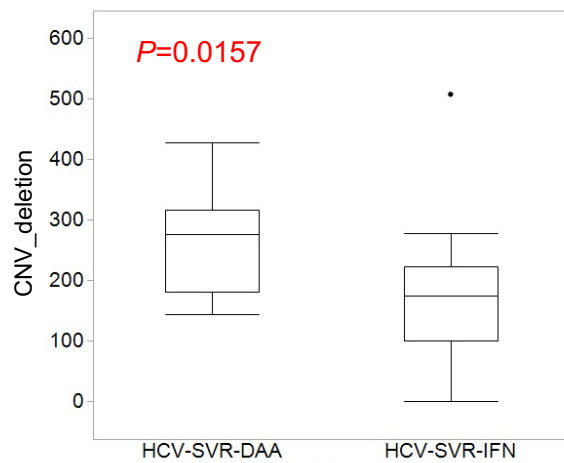**c**

Supplement: Supplementary file 6 — Figure S6 [file CAM4-11-1769-s008.pdf]

Present study cohort

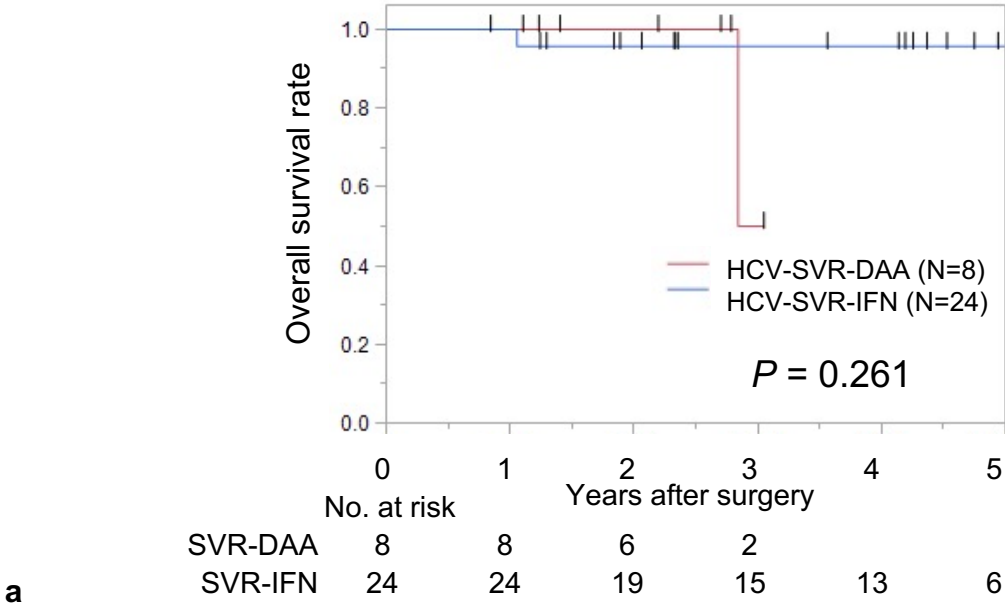

Present study cohort

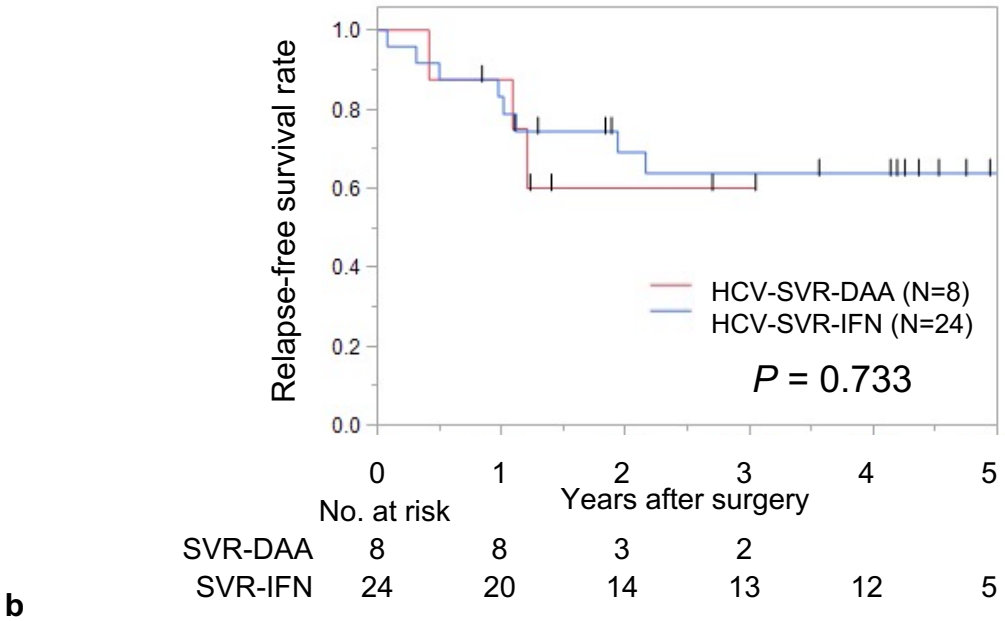

TCGA liver cancer cohort

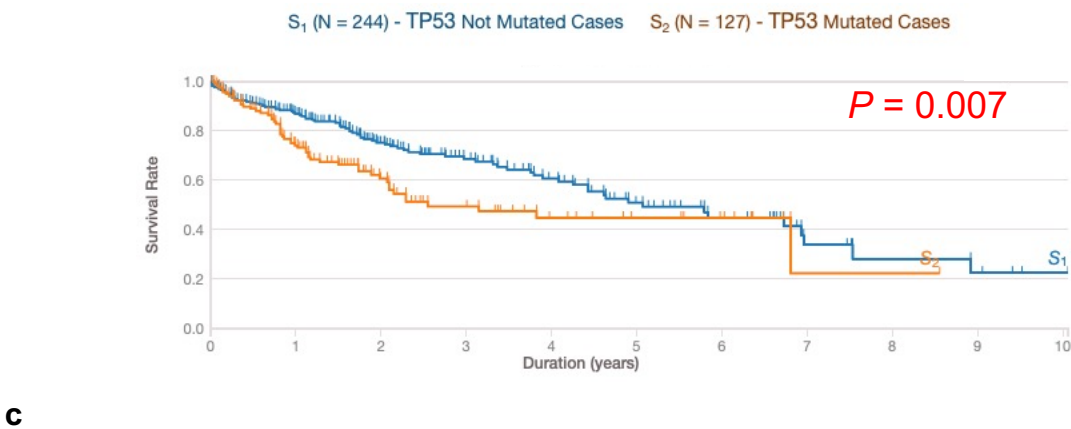

Supplement: Supplementary file 7 — Figure S7 [file CAM4-11-1769-s007.pdf]
