# Supplementary material for: Hepatocellular carcinoma after a sustained virological response by direct‐acting antivirals harbors TP53 inactivation
Source: Cancer Med. 2022 Feb 17;11(8):1769–86. doi: 10.1002/cam4.4571 (PMC9041076; doi:10.1002/cam4.4571)
Supplement: Supplementary file 4 — Figure S4 [file CAM4-11-1769-s003.pdf]

ROBO1 mRNA expression

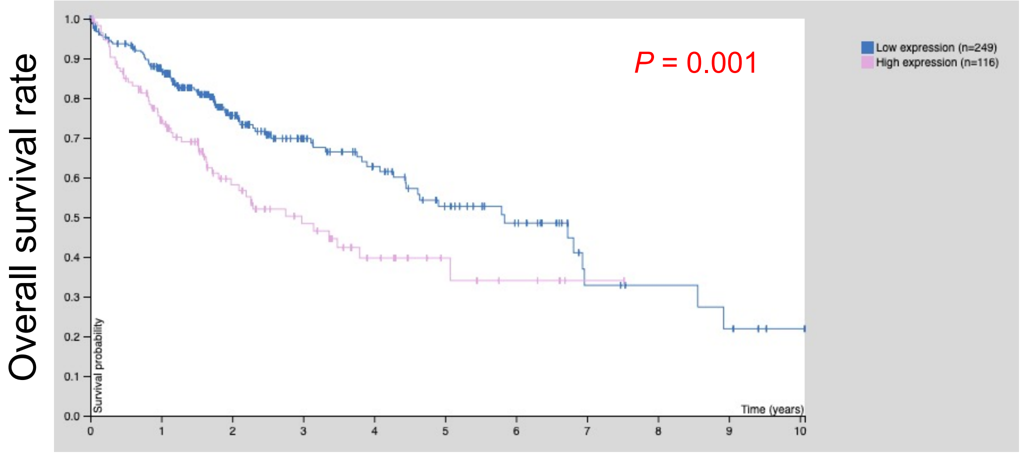

Years after surgery

COL22A1 mRNA expression

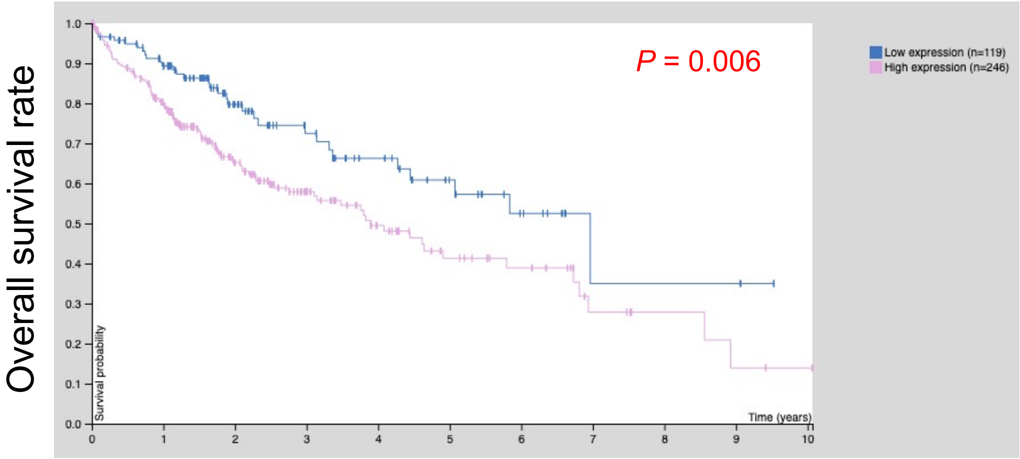

Years after surgery

SRGAP3 mRNA expression

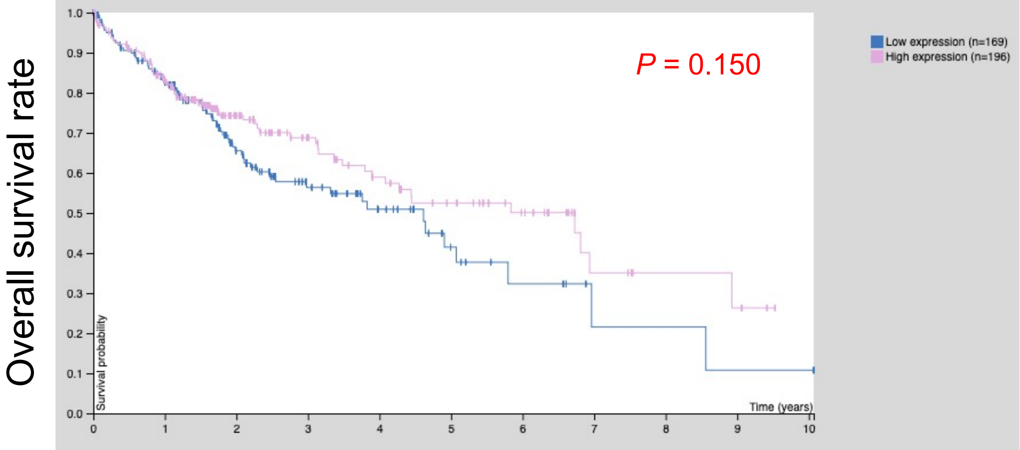

Years after surgery
