# Supplementary material for: Hepatocellular carcinoma after a sustained virological response by direct‐acting antivirals harbors TP53 inactivation
Source: Cancer Med. 2022 Feb 17;11(8):1769–86. doi: 10.1002/cam4.4571 (PMC9041076; doi:10.1002/cam4.4571)
Supplement: Supplementary file 8 — Table S1 [file CAM4-11-1769-s006.pdf]

Supplementary Table S2. TCGA data list used in this study.

Barcode	MutSig exome analysis	Gene expression analysis
TCGA-2V-A95S-01	1	0
TCGA-2Y-A9GS-01	1	1
TCGA-2Y-A9GT-01	1	1
TCGA-2Y-A9GU-01	1	1
TCGA-2Y-A9GV-01	1	1
TCGA-2Y-A9GW-01	1	1
TCGA-2Y-A9GX-01	1	1
TCGA-2Y-A9GY-01	1	1
TCGA-2Y-A9GZ-01	1	1
TCGA-2Y-A9H0-01	1	1
TCGA-2Y-A9H1-01	1	1
TCGA-2Y-A9H2-01	1	1
TCGA-2Y-A9H3-01	1	1
TCGA-2Y-A9H4-01	1	1
TCGA-2Y-A9H5-01	1	1
TCGA-2Y-A9H6-01	1	1
TCGA-2Y-A9H7-01	1	1
TCGA-2Y-A9H8-01	1	1
TCGA-2Y-A9H9-01	1	1
TCGA-2Y-A9HA-01	1	1
TCGA-2Y-A9HB-01	1	1
TCGA-3K-AAZ8-01	1	1
TCGA-4R-AA8I-01	1	1
TCGA-5C-A9VG-01	1	1
TCGA-5C-A9VH-01	1	1
TCGA-5C-AAPD-01	1	1
TCGA-5R-AA1C-01	1	1
TCGA-5R-AA1D-01	1	1
TCGA-5R-AAAM-01	1	1
TCGA-BC-4072-01	0	1
TCGA-BC-4073-01	1	1
TCGA-BC-A10Q-01	1	1
TCGA-BC-A10R-01	1	1
TCGA-BC-A10S-01	0	1
TCGA-BC-A10T-01	1	1
TCGA-BC-A10U-01	1	1
TCGA-BC-A10W-01	1	1
TCGA-BC-A10X-01	1	1
TCGA-BC-A10Y-01	1	1
TCGA-BC-A10Z-01	1	1
TCGA-BC-A110-01	0	1
TCGA-BC-A112-01	0	1
TCGA-BC-A216-01	1	1
TCGA-BC-A217-01	1	1
TCGA-BC-A3KF-01	1	1
TCGA-BC-A3KG-01	0	1
TCGA-BC-A5W4-01	1	1
TCGA-BC-A69H-01	1	1
TCGA-BC-A69I-01	0	1
TCGA-BC-A8YO-01	1	1
TCGA-BD-A2L6-01	1	1
TCGA-BD-A3EP-01	1	1
TCGA-BD-A3ER-01	0	1
TCGA-BW-A5NO-01	1	1
TCGA-BW-A5NP-01	1	0
TCGA-BW-A5NQ-01	1	0
TCGA-CC-5258-01	1	1
TCGA-CC-5259-01	1	1
TCGA-CC-5260-01	1	1
TCGA-CC-5261-01	0	1
TCGA-CC-5262-01	1	1
TCGA-CC-5263-01	1	1
TCGA-CC-5264-01	1	1
TCGA-CC-A123-01	1	1
TCGA-CC-A1HT-01	1	1
TCGA-CC-A3M9-01	1	1
TCGA-CC-A3MA-01	1	1
TCGA-CC-A3MB-01	1	1
TCGA-CC-A3MC-01	1	1
TCGA-CC-A5UC-01	1	1
TCGA-CC-A5UD-01	1	1
TCGA-CC-A5UE-01	1	1
TCGA-CC-A7IE-01	1	1
TCGA-CC-A7IF-01	1	1
TCGA-CC-A7IG-01	1	1
TCGA-CC-A7IH-01	1	1
TCGA-CC-A7II-01	1	1
TCGA-CC-A7IJ-01	1	1
TCGA-CC-A7IK-01	1	1
TCGA-CC-A7IL-01	1	1
TCGA-CC-A8HS-01	1	1
TCGA-CC-A8HT-01	1	1
TCGA-CC-A8HU-01	1	1
TCGA-CC-A8HV-01	1	1
TCGA-CC-A9FS-01	1	1
TCGA-CC-A9FU-01	1	0
TCGA-CC-A9FV-01	1	0
TCGA-CC-A9FW-01	1	1
TCGA-DD-A113-01	1	1
TCGA-DD-A114-01	1	1
TCGA-DD-A115-01	1	1
TCGA-DD-A116-01	0	1
TCGA-DD-A118-01	1	1
TCGA-DD-A119-01	1	1
TCGA-DD-A11A-01	0	1
TCGA-DD-A11B-01	1	1
TCGA-DD-A11C-01	1	1
TCGA-DD-A11D-01	1	1
TCGA-DD-A1E9-01	1	0
TCGA-DD-A1EA-01	0	1
TCGA-DD-A1EB-01	0	1
TCGA-DD-A1EC-01	1	1
TCGA-DD-A1ED-01	1	1
TCGA-DD-A1EE-01	0	1
TCGA-DD-A1EF-01	0	1
TCGA-DD-A1EG-01	0	1
TCGA-DD-A1EH-01	0	1
TCGA-DD-A1EI-01	1	1
TCGA-DD-A1EJ-01	1	1
TCGA-DD-A1EK-01	1	1
TCGA-DD-A1EL-01	1	1
TCGA-DD-A39V-01	1	1
TCGA-DD-A39W-01	1	1
TCGA-DD-A39X-01	1	1
TCGA-DD-A39Y-01	0	1
TCGA-DD-A39Z-01	1	1
TCGA-DD-A3A0-01	0	0
TCGA-DD-A3A1-01	0	1
TCGA-DD-A3A2-01	1	1
TCGA-DD-A3A3-01	1	1
TCGA-DD-A3A4-01	1	1
TCGA-DD-A3A5-01	1	1
TCGA-DD-A3A6-01	1	1
TCGA-DD-A3A7-01	1	1
TCGA-DD-A3A8-01	1	1
TCGA-DD-A3A9-01	1	1

TCGA-DD-A4NA-01	1	1
TCGA-DD-A4NB-01	1	1
TCGA-DD-A4ND-01	1	1
TCGA-DD-A4NE-01	1	1
TCGA-DD-A4NF-01	1	1
TCGA-DD-A4NG-01	1	1
TCGA-DD-A4NH-01	1	1
TCGA-DD-A4NI-01	1	1
TCGA-DD-A4NJ-01	1	1
TCGA-DD-A4NK-01	1	1
TCGA-DD-A4NL-01	1	1
TCGA-DD-A4NN-01	1	1
TCGA-DD-A4NO-01	1	1
TCGA-DD-A4NP-01	1	1
TCGA-DD-A4NQ-01	1	1
TCGA-DD-A4NR-01	1	1
TCGA-DD-A4NS-01	1	1
TCGA-DD-A4NV-01	1	1
TCGA-DD-A73A-01	1	1
TCGA-DD-A73B-01	1	1
TCGA-DD-A73C-01	1	1
TCGA-DD-A73D-01	1	1
TCGA-DD-A73E-01	1	1
TCGA-DD-A73F-01	1	1
TCGA-DD-A73G-01	1	1
TCGA-DD-AA3A-01	1	1
TCGA-DD-AAC8-01	1	1
TCGA-DD-AAC9-01	1	1
TCGA-DD-AACA-01	1	1
TCGA-DD-AACA-02	0	0
TCGA-DD-AACB-01	1	1
TCGA-DD-AACC-01	1	1
TCGA-DD-AACD-01	1	1
TCGA-DD-AACE-01	1	1
TCGA-DD-AACF-01	1	1
TCGA-DD-AACG-01	1	1
TCGA-DD-AACH-01	1	1
TCGA-DD-AACI-01	1	1
TCGA-DD-AACJ-01	1	1
TCGA-DD-AACK-01	1	1
TCGA-DD-AACL-01	1	1
TCGA-DD-AACM-01	1	0
TCGA-DD-AACN-01	1	1
TCGA-DD-AACO-01	1	1
TCGA-DD-AACP-01	1	1
TCGA-DD-AACQ-01	1	1
TCGA-DD-AACS-01	1	1
TCGA-DD-AACT-01	1	1
TCGA-DD-AACU-01	1	1
TCGA-DD-AACV-01	1	1
TCGA-DD-AACW-01	1	1
TCGA-DD-AACX-01	1	1
TCGA-DD-AACY-01	1	1
TCGA-DD-AACZ-01	1	1
TCGA-DD-AAD0-01	1	1
TCGA-DD-AAD1-01	1	1
TCGA-DD-AAD2-01	1	1
TCGA-DD-AAD3-01	1	1
TCGA-DD-AAD5-01	1	1
TCGA-DD-AAD6-01	1	1
TCGA-DD-AAD8-01	1	1
TCGA-DD-AADA-01	1	1
TCGA-DD-AADB-01	1	1
TCGA-DD-AADC-01	1	1
TCGA-DD-AADD-01	1	1
TCGA-DD-AADE-01	1	0
TCGA-DD-AADF-01	1	1
TCGA-DD-AADG-01	1	1
TCGA-DD-AADI-01	1	1
TCGA-DD-AADJ-01	1	1
TCGA-DD-AADK-01	1	1
TCGA-DD-AADL-01	1	1
TCGA-DD-AADM-01	1	1
TCGA-DD-AADN-01	1	1
TCGA-DD-AADO-01	1	1
TCGA-DD-AADP-01	1	1
TCGA-DD-AADQ-01	1	1
TCGA-DD-AADR-01	1	1
TCGA-DD-AADS-01	1	1
TCGA-DD-AADU-01	1	1
TCGA-DD-AADV-01	1	1
TCGA-DD-AADW-01	1	1
TCGA-DD-AADY-01	1	1
TCGA-DD-AAE0-01	1	1
TCGA-DD-AAE1-01	1	1
TCGA-DD-AAE2-01	1	1
TCGA-DD-AAE3-01	1	1
TCGA-DD-AAE4-01	1	1
TCGA-DD-AAE6-01	1	1
TCGA-DD-AAE7-01	1	1
TCGA-DD-AAE8-01	1	0
TCGA-DD-AAE9-01	1	1
TCGA-DD-AAEA-01	1	1
TCGA-DD-AAEB-01	1	1
TCGA-DD-AAED-01	1	1
TCGA-DD-AAEE-01	1	1
TCGA-DD-AAEG-01	1	1
TCGA-DD-AAEH-01	1	1
TCGA-DD-AAEI-01	1	1
TCGA-DD-AAEK-01	1	1
TCGA-DD-AAVP-01	1	1
TCGA-DD-AAVQ-01	1	1
TCGA-DD-AAVR-01	1	1
TCGA-DD-AAVS-01	1	1
TCGA-DD-AAVU-01	1	1
TCGA-DD-AAVV-01	1	1
TCGA-DD-AAVW-01	1	1
TCGA-DD-AAVX-01	1	1
TCGA-DD-AAVY-01	1	1
TCGA-DD-AAVZ-01	1	1
TCGA-DD-AAW0-01	1	1
TCGA-DD-AAW1-01	1	1
TCGA-DD-AAW2-01	1	1
TCGA-DD-AAW3-01	1	1
TCGA-ED-A459-01	1	1
TCGA-ED-A4XI-01	1	1
TCGA-ED-A5KG-01	1	1
TCGA-ED-A627-01	1	1
TCGA-ED-A66X-01	1	1
TCGA-ED-A66Y-01	1	1
TCGA-ED-A7PX-01	1	1
TCGA-ED-A7PY-01	1	1
TCGA-ED-A7PZ-01	1	1
TCGA-ED-A7XO-01	1	1
TCGA-ED-A7XP-01	1	1
TCGA-ED-A82E-01	1	1
TCGA-ED-A8O5-01	1	1
TCGA-ED-A8O6-01	1	1
TCGA-ED-A97K-01	1	1
TCGA-EP-A12J-01	0	1
TCGA-EP-A26S-01	1	1

TCGA-EP-A2KA-01	1	1
TCGA-EP-A2KB-01	1	1
TCGA-EP-A2KC-01	1	1
TCGA-EP-A3JL-01	1	1
TCGA-EP-A3RK-01	1	1
TCGA-ES-A2HS-01	1	1
TCGA-ES-A2HT-01	1	1
TCGA-FV-A23B-01	1	1
TCGA-FV-A2QQ-01	1	1
TCGA-FV-A2QR-01	1	1
TCGA-FV-A3I0-01	1	1
TCGA-FV-A3I1-01	1	1
TCGA-FV-A3R2-01	1	1
TCGA-FV-A3R3-01	1	1
TCGA-FV-A495-01	1	1
TCGA-FV-A496-01	1	1
TCGA-FV-A4ZP-01	1	1
TCGA-FV-A4ZQ-01	1	1
TCGA-G3-A25S-01	1	1
TCGA-G3-A25T-01	1	1
TCGA-G3-A25U-01	1	1
TCGA-G3-A25V-01	1	1
TCGA-G3-A25W-01	1	0
TCGA-G3-A25X-01	0	1
TCGA-G3-A25Y-01	1	1
TCGA-G3-A25Z-01	1	1
TCGA-G3-A3CG-01	0	1
TCGA-G3-A3CH-01	1	1
TCGA-G3-A3CI-01	1	1
TCGA-G3-A3CJ-01	0	1
TCGA-G3-A3CK-01	1	1
TCGA-G3-A5SI-01	1	1
TCGA-G3-A5SJ-01	1	1
TCGA-G3-A5SK-01	1	1
TCGA-G3-A5SL-01	1	1
TCGA-G3-A5SM-01	1	1
TCGA-G3-A6UC-01	1	1
TCGA-G3-A7M5-01	1	1
TCGA-G3-A7M6-01	1	1
TCGA-G3-A7M7-01	1	1
TCGA-G3-A7M8-01	1	1
TCGA-G3-A7M9-01	1	1
TCGA-G3-AAUZ-01	1	1
TCGA-G3-AAV0-01	1	1
TCGA-G3-AAV1-01	1	1
TCGA-G3-AAV2-01	1	1
TCGA-G3-AAV3-01	1	1
TCGA-G3-AAV4-01	1	1
TCGA-G3-AAV5-01	1	1
TCGA-G3-AAV6-01A	1	1
TCGA-G3-AAV7-01	1	1
TCGA-GJ-A3OU-01	1	1
TCGA-GJ-A6C0-01	1	1
TCGA-GJ-A9DB-01	1	1
TCGA-HP-A5MZ-01	1	1
TCGA-HP-A5N0-01	1	1
TCGA-K7-A5RF-01	1	1
TCGA-K7-A5RG-01	1	1
TCGA-K7-A6G5-01	1	1
TCGA-K7-AAU7-01	1	1
TCGA-KR-A7K0-01	1	1
TCGA-KR-A7K2-01	1	1
TCGA-KR-A7K7-01	1	1
TCGA-KR-A7K8-01	1	1
TCGA-LG-A6GG-01	1	1
TCGA-LG-A9QC-01	1	1
TCGA-LG-A9QD-01	1	1
TCGA-MI-A75C-01	1	1
TCGA-MI-A75E-01	1	1
TCGA-MI-A75G-01	1	1
TCGA-MI-A75H-01	1	1
TCGA-MI-A75I-01	1	1
TCGA-MR-A520-01	1	1
TCGA-MR-A8JO-01	1	1
TCGA-NI-A4U2-01	1	1
TCGA-NI-A8LF-01	1	1
TCGA-O8-A75V-01	1	1
TCGA-PD-A5DF-01	1	1
TCGA-QA-A7B7-01	1	1
TCGA-RC-A6M3-01	1	0
TCGA-RC-A6M4-01	1	1
TCGA-RC-A6M5-01	1	1
TCGA-RC-A6M6-01	1	1
TCGA-RC-A7S9-01	1	1
TCGA-RC-A7SB-01	1	1
TCGA-RC-A7SF-01	1	1
TCGA-RC-A7SH-01	1	1
TCGA-RC-A7SK-01	1	1
TCGA-RG-A7D4-01	1	1
TCGA-T1-A6J8-01	0	1
TCGA-UB-A7MA-01	1	1
TCGA-UB-A7MB-01	1	1
TCGA-UB-A7MC-01	1	1
TCGA-UB-A7MD-01	1	1
TCGA-UB-A7ME-01	1	1
TCGA-UB-A7MF-01	1	1
TCGA-UB-AA0U-01	1	1
TCGA-UB-AA0V-01	1	1
TCGA-WJ-A86L-01	1	1
TCGA-WQ-A9G7-01	1	1
TCGA-WQ-AB4B-01	1	1
TCGA-WX-AA44-01	1	1
TCGA-WX-AA46-01	1	1
TCGA-WX-AA47-01	1	1
TCGA-XR-A8TC-01	1	1
TCGA-XR-A8TD-01	1	1
TCGA-XR-A8TE-01	1	1
TCGA-XR-A8TF-01	1	1
TCGA-XR-A8TG-01	1	1
TCGA-YA-A8S7-01	1	1
TCGA-ZP-A9CV-01	1	1
TCGA-ZP-A9CY-01	1	1
TCGA-ZP-A9CZ-01	1	1
TCGA-ZP-A9D0-01	1	1
TCGA-ZP-A9D1-01	1	1
TCGA-ZP-A9D2-01	1	1
TCGA-ZP-A9D4-01	1	1
TCGA-ZS-A9CD-01	1	1
TCGA-ZS-A9CE-01	1	1
TCGA-ZS-A9CF-01	1	1
TCGA-ZS-A9CF-02	0	0
TCGA-ZS-A9CG-01	1	1
TCGA-BD-A3ER-01	1	0
TCGA-CC-A8HS-01	1	0
TCGA-DD-AACA-02	1	0
TCGA-G3-A3CG-01	1	0
TCGA-G3-AAV6-01A	1	0
TCGA-T1-A6J8-01	1	0
TCGA-XR-A8TC-01	1	0
TCGA-ZS-A9CF-02	1	0
