# Supplementary material for: Hepatocellular carcinoma after a sustained virological response by direct‐acting antivirals harbors TP53 inactivation
Source: Cancer Med. 2022 Feb 17;11(8):1769–86. doi: 10.1002/cam4.4571 (PMC9041076; doi:10.1002/cam4.4571)
Supplement: Supplementary file 9 — Table S2 [file CAM4-11-1769-s009.pdf]

Supplementary Table S2. Gene expression signatures used in this study

Gene expression signature	Cytolytic activity	CD8_T_cells	NK_cells	B_cells	IFN_alpha_response	IFN_gamma_response	Wnt/β-catenin signaling	TP53 inactivation score	CIN signature	PI3K/mTOR CMAP UP signature	T cell-inflamed GEP signature
Reference	Ref. 17	Ref. 18	Ref. 18	Ref. 18	Ref. 19	Ref. 19	Ref. 19	Ref. 20	Ref. 21-23	Ref. 24-26	Ref. 27-29
Gene symbols	*PRF1*<br>*GZMA*	*ABT1*<br>*AES*<br>*APBA2*<br>*ARHGAP8*<br>*C12orf47*<br>*C19orf6*<br>*C4orf15*<br>*CAMLG*<br>*CD8A*<br>*CD8B*<br>*CDKN2AIP*<br>*DNAJB1*<br>*FLT3LG*<br>*GADD45A*<br>*GZMM*<br>*KLF9*<br>*LEPROTL1*<br>*LIME1*<br>*MYST3*<br>*PF4*<br>*PPP1R2*<br>*PRF1*<br>*PRR5*<br>*RBM3*<br>*SF1*<br>*SFRS7*<br>*SLC16A7*<br>*TBCC*<br>*THUMPD1*<br>*TMC6*<br>*TSC22D3*<br>*VAMP2*<br>*ZEB1*<br>*ZFP36L2*<br>*ZNF22*<br>*ZNF609*<br>*ZNF91*	*ADARB1*<br>*AF107846*<br>*AL080130*<br>*ALDH1B1*<br>*ARL6IP2*<br>*BCL2*<br>*CDC5L*<br>*FGF18*<br>*FUT5*<br>*FZR1*<br>*IGFBP5*<br>*LDB3*<br>*MAPRE3*<br>*MCM3AP*<br>*MRC2*<br>*NCR1*<br>*NM_014274*<br>*PDLIM4*<br>*PRX*<br>*PSMD4*<br>*SLC30A5*<br>*SMEK1*<br>*SPN*<br>*TBXA2R*<br>*TCTN2*<br>*TINAGL1*<br>*XCL1*<br>*ZNF205*<br>*ZNF528*<br>*ZNF747*	*ABCB4*<br>*BACH2*<br>*BCL11A*<br>*BLK*<br>*BLNK*<br>*CCR9*<br>*CD19*<br>*CD72*<br>*COCH*<br>*CR2*<br>*DTNB*<br>*FAM30A*<br>*FCRL2*<br>*GLDC*<br>*GNG7*<br>*HLA-DOB*<br>*HLA-DQA1*<br>*KIAA0125*<br>*MEF2C*<br>*MICAL3*<br>*MS4A1*<br>*OSBPL10*<br>*PNOC*<br>*QRSL1*<br>*SCN3A*<br>*SLC15A2*<br>*SPIB*<br>*TCL1A*<br>*TNFRSF17*	*ADAR*<br>*B2M*<br>*BATF2*<br>*BST2*<br>*C1S*<br>*CASP1*<br>*CASP8*<br>*CCRL2*<br>*CD47*<br>*CD74*<br>*CMPK2*<br>*CNP*<br>*CSF1*<br>*CXCL10*<br>*CXCL11*<br>*DDX60*<br>*DHX58*<br>*EIF2AK2*<br>*ELF1*<br>*EPSTI1*<br>*FAM125A*<br>*FAM46A*<br>*FTSJD2*<br>*GBP2*<br>*GBP4*<br>*GMPR*<br>*HERC6*<br>*HLA-C*<br>*IFI27*<br>*IFI30*<br>*IFI35*<br>*IFI44*<br>*IFI44L*<br>*IFIH1*<br>*IFIT2*<br>*IFIT3*<br>*IFITM1*<br>*IFITM2*<br>*IFITM3*<br>*IL15*<br>*IL4R*<br>*IL7*<br>*IRF1*<br>*IRF2*<br>*IRF7*<br>*IRF9*<br>*ISG15*<br>*ISG20*<br>*LAMP3*<br>*LAP3*<br>*LGALS3BP*<br>*LPAR6*<br>*LY6E*<br>*MOV10*<br>*MX1*<br>*NCOA7*<br>*NMI*<br>*NUB1*<br>*OAS1*<br>*OASL*<br>*OGFR*<br>*PARP12*<br>*PARP14*<br>*PARP9*<br>*PLSCR1*<br>*PNPT1*<br>*PRIC285*<br>*PROCR*<br>*PSMA3*<br>*PSMB8*<br>*PSMB9*<br>*PSME1*<br>*PSME2*<br>*RIPK2*<br>*RNF31*<br>*RSAD2*<br>*RTP4*<br>*SAMD9*<br>*SAMD9L*<br>*SELL*<br>*SLC25A28*<br>*SP110*<br>*STAT2*<br>*TAP1*<br>*TDRD7*<br>*TMEM140*<br>*TRAFD1*<br>*TRIM14*<br>*TRIM21*<br>*TRIM25*<br>*TRIM26*<br>*TRIM5*<br>*TXNIP*<br>*UBA7*<br>*UBE2L6*<br>*USP18*<br>*WARS*	*AIF1*<br>*AQP4*<br>*ASS1*<br>*B2M*<br>*CAMK2A*<br>*CAMK2B*<br>*CAMK2D*<br>*CAMK2G*<br>*CCL1*<br>*CCL11*<br>*CCL13*<br>*CCL14*<br>*CCL15*<br>*CCL16*<br>*CCL17*<br>*CCL18*<br>*CCL19*<br>*CCL2*<br>*CCL20*<br>*CCL21*<br>*CCL22*<br>*CCL23*<br>*CCL24*<br>*CCL25*<br>*CCL26*<br>*CCL3*<br>*CCL3L3*<br>*CCL4*<br>*CCL4L2*<br>*CCL5*<br>*CCL7*<br>*CCL8*<br>*CD44*<br>*CD58*<br>*CIITA*<br>*CX3CL1*<br>*DAPK1*<br>*DAPK3*<br>*EDN1*<br>*EPRS*<br>*FCGR1A*<br>*FCGR1B*<br>*GAPDH*<br>*GBP1*<br>*GBP2*<br>*GBP5*<br>*GBP6*<br>*HCK*<br>*HLA-A*<br>*HLA-B*<br>*HLA-C*<br>*HLA-DPA1*<br>*HLA-DPB1*<br>*HLA-DQA1*<br>*HLA-DQA2*<br>*HLA-DQB1*<br>*HLA-DQB2*<br>*HLA-DRA*<br>*HLA-DRB1*<br>*HLA-DRB3*<br>*HLA-DRB4*<br>*HLA-DRB5*<br>*HLA-E*<br>*HLA-F*<br>*HLA-G*<br>*ICAM1*<br>*IFI30*<br>*IFNG*<br>*IFNGR1*<br>*IFNGR2*<br>*IL12B*<br>*IL12RB1*<br>*IRF1*<br>*IRF2*<br>*IRF3*<br>*IRF4*<br>*IRF5*<br>*IRF6*<br>*IRF7*<br>*IRF8*<br>*IRF9*<br>*IRG1*<br>*JAK1*<br>*JAK2*<br>*LGALS9*<br>*MID1*<br>*MRC1*<br>*MT2A*<br>*NCAM1*<br>*NOS2*<br>*OAS1*<br>*OAS2*<br>*OAS3*<br>*OASL*<br>*PML*<br>*PRKCD*<br>*PTAFR*<br>*RPL13A*<br>*SLC26A6*<br>*SP100*<br>*STAR*<br>*STAT1*<br>*SYNCRIP*<br>*TDGF1*<br>*TLR3*<br>*TRIM21*<br>*TRIM22*<br>*TRIM25*<br>*TRIM26*<br>*TRIM31*<br>*TRIM34*<br>*TRIM38*<br>*TRIM5*<br>*TRIM62*<br>*TRIM68*<br>*TRIM8*<br>*VCAM1*<br>*WNT5A*<br>*XCL1*	*AADAC*<br>*ABCB11*<br>*ABCG2*<br>*ABHD6*<br>*ACE2*<br>*ACSL5*<br>*ACSL6*<br>*ACSM3*<br>*ACSS3*<br>*ACTN2*<br>*ADH6*<br>*ADRB2*<br>*ADRBK2*<br>*ALDH1L1*<br>*ALDH3A1*<br>*ALDH3A2*<br>*AMACR*<br>*ANKFN1*<br>*AOX1*<br>*AQP11*<br>*AQP6*<br>*AQP9*<br>*AR*<br>*ASAP2*<br>*ASPSCR1*<br>*AXIN2*<br>*BAMBI*<br>*BIK*<br>*BMP4*<br>*BOK*<br>*C1orf112*<br>*C1orf53*<br>*C6orf97*<br>*C9orf5*<br>*CAP2*<br>*CCDC53*<br>*CD36*<br>*CDC14B*<br>*CDK6*<br>*CLDN2*<br>*CORIN*<br>*CPPED1*<br>*CRLS1*<br>*CST1*<br>*CTNNA2*<br>*CTNNBL1*<br>*CYP1A1*<br>*CYP2E1*<br>*CYP8B1*<br>*DCXR*<br>*DNAJC12*<br>*DPP4*<br>*DSG1*<br>*DYNC1I1*<br>*EBPL*<br>*ECM2*<br>*EPHB2*<br>*ESRRG*<br>*EXPH5*<br>*FAM169A*<br>*FAM35A*<br>*FAM3B*<br>*FAM8A1*<br>*FAS*<br>*FGF13*<br>*FIGF*<br>*FITM2*<br>*FLJ22763*<br>*FRMD3*<br>*GFRA1*<br>*GLUL*<br>*GLYAT*<br>*GNAI1*<br>*GPAM*<br>*GPHN*<br>*GRHPR*<br>*GSTM2*<br>*HABP4*<br>*HEPACAM*<br>*HHAT*<br>*HIBADH*<br>*HIST1H2AE*<br>*HLF*<br>*HOGA1*<br>*HPD*<br>*HRASLS2*<br>*HSD11B1*<br>*HSDL2*<br>*HTR2B*<br>*INSIG2*<br>*IRS1*<br>*IRX3*<br>*ITPR2*<br>*KCNJ8*<br>*KCNK1*<br>*LGR5*<br>*LINC00176*<br>*LPPR1*<br>*MAP3K8*<br>*MERTK*<br>*MME*<br>*MTHFD1*<br>*MYRIP*<br>*NAGS*<br>*NEK3*<br>*NKD1*<br>*NUBPL*<br>*NUDT6*<br>*ODAM*<br>*ODZ2*<br>*PAGE4*<br>*PANX1*<br>*PDK1*<br>*PDK4*<br>*PHLPP1*<br>*PHYHIPL*<br>*PREB*<br>*PRR5L*<br>*PTPRG*<br>*RAB11FIP2*<br>*RBP1*<br>*REG1A*<br>*REG3A*<br>*RHBG*<br>*RHOBTB1*<br>*RTP3*<br>*RUNDC3B*<br>*SALL1*<br>*SDPR*<br>*SEC14L2*<br>*SELENBP1*<br>*SEPT4*<br>*SGK223*<br>*SLC13A3*<br>*SLC16A1*<br>*SLC16A10*<br>*SLC16A11*<br>*SLC16A4*<br>*SLC17A1*<br>*SLC1A2*<br>*SLC22A11*<br>*SLC22A4*<br>*SLC25A30*<br>*SLC2A12*<br>*SLC47A1*<br>*SLC4A4*<br>*SLC5A6*<br>*SLC6A12*<br>*SLCO1B1*<br>*SMPX*<br>*SNAI2*<br>*SPARCL1*<br>*SPRYD7*<br>*SRD5A2*<br>*STRA13*<br>*SULT1B1*<br>*TBCK*<br>*TBX3*<br>*THBS4*<br>*TMEM100*<br>*TMEM64*<br>*TNFRSF19*<br>*TPRG1*<br>*TRIB2*<br>*TSPAN5*<br>*TTC30A*<br>*TTC9*<br>*TTPA*<br>*UBXN10*<br>*UST*<br>*VLDLR*<br>*YPEL1*<br>*ZNF385B*<br>*ZNRF3*	*CDC20*<br>*PLK1*<br>*CENPA*<br>*KIF2C*	*ACTL6A*<br>*ELAVL1*<br>*MAD2L1*<br>*NEK2*<br>*OIP5*	*AARS*<br>*ABCF1*<br>*ACAT2*<br>*ACLY*<br>*ATP1B1*<br>*ATP6V0B*<br>*CALU*<br>*CASP7*<br>*CBR1*<br>*CCND3*<br>*CSK*<br>*CSRP1*<br>*DDX10*<br>*DFFA*<br>*DUSP3*<br>*FDFT1*<br>*FHL2*<br>*FKBP4*<br>*GABPB1*<br>*GALE*<br>*GLI2*<br>*GLRX*<br>*GNA11*<br>*GRB10*<br>*MSH6*<br>*GTF2A2*<br>*GTF2E2*<br>*HDAC2*<br>*HIF1A*<br>*HMGCR*<br>*HMGCS1*<br>*HMOX1*<br>*HPRT1*<br>*HSPA1A*<br>*HSPA4*<br>*HSPD1*<br>*DNAJB1*<br>*ICAM3*<br>*ITGAE*<br>*KIF5C*<br>*MAT2A*<br>*MCM3*<br>*PNP*<br>*NRAS*<br>*NUCB2*<br>*NUP88*<br>*PAFAH1B1*<br>*PMAIP1*<br>*PMM2*<br>*POLE2*<br>*PPIC*<br>*RFC5*<br>*RPN1*<br>*SPR*<br>*DYNLT3*<br>*TFDP1*<br>*TPD52L2*<br>*TXNRD1*<br>*UGDH*<br>*MAPKAPK3*<br>*FOSL1*<br>*BHLHE40*<br>*PSMG1*<br>*IER3*<br>*DNAJA3*<br>*USP14*<br>*PRPF4*<br>*CCNE2*<br>*VAPB*<br>*UBE2L6*<br>*ITGB1BP1*<br>*HERPUD1*<br>*MLEC*<br>*TATDN2*<br>*MRPL19*<br>*LPGAT1*<br>*PARP2*<br>*G3BP1*<br>*TXNDC9*<br>*SLC35B1*<br>*HYOU1*<br>*DRAP1*<br>*EBP*<br>*WASF3*<br>*UTP14A*<br>*BLCAP*<br>*TCERG1*<br>*MLLT11*<br>*CCDC85B*<br>*TOPBP1*<br>*CORO1A*<br>*SLC2A6*<br>*NISCH*<br>*ECD*<br>*PUF60*<br>*RPIA*<br>*RRP1B*<br>*JMJD6*<br>*RRS1*<br>*ARFIP2*<br>*RAI14*<br>*TES*<br>*PHGDH*<br>*TRAPPC3*<br>*DNTTIP2*<br>*GMNN*<br>*ATP6V1D*<br>*PRKAG2*<br>*UBE2J1*<br>*GFOD1*<br>*KCTD5*<br>*EXOSC4*<br>*DDIT4*<br>*YTHDF1*<br>*FKBP14*<br>*HEATR1*<br>*ANO10*<br>*TRIB3*<br>*ENOPH1*<br>*ATG3*<br>*ZDHHC6*<br>*ACD*<br>*TMEM109*<br>*CCDC86*<br>*CHAC1*<br>*CRELD2*<br>*CHMP6*<br>*NUP85*<br>*DHDDS*<br>*GRWD1*<br>*TUBB6*<br>*MICALL1*<br>*H2AFV*<br>*RPL39L*	*TIGIT*<br>*CD27*<br>*CD8A*<br>*PDCD1LG2*<br>*LAG3*<br>*CD274*<br>*CXCR6*<br>*CMKLR1*<br>*NKG7*<br>*CCL5*<br>*PSMB10*<br>*IDO1*<br>*CXCL9*<br>*HLA-DQA1*<br>*CD276*<br>*STAT1*<br>*HLA-DRB1*<br>*HLA-E*
